# Explaining naturalization and invasiveness: new insights from historical ornamental plant catalogs

**DOI:** 10.1002/ece3.2471

**Published:** 2016-09-15

**Authors:** Claude Lavoie, Simon Joly, Alexandre Bergeron, Geneviève Guay, Elisabeth Groeneveld

**Affiliations:** ^1^ École Supérieure d'Aménagement du Territoire et de Développement Régional and Centre de la Science de la Biodiversité du Québec Université Laval Québec City QC Canada; ^2^ Institut de Recherche en Biologie Végétale Université de Montréal Montréal QC Canada; ^3^ Jardin Botanique de Montréal Montréal QC Canada

**Keywords:** climate warming, cold‐temperate region, herbarium specimen, ornamental plant, phylogeny, plant hardiness zone, plant invasion

## Abstract

We identified plant attributes associated with naturalization and invasiveness using century‐old ornamental plant catalogs from Québec (Canada). We tested the hypothesis that naturalization is determined by fewer factors than invasiveness, as the latter also requires dispersal, which introduces additional complexity. The approach we used took into account not only plant attributes as explanatory factors, but also propagule pressure, while accounting for phylogenetic relationships among species. Museum collections were used, in combination with scientific journal databases, to assess invasiveness. Particular attention was given to species that never escaped from gardens and thus represent cases of “failed” invasions. Naturalization in cold‐temperate environments is determined by fewer factors than invasion, but only if phylogenetic links between species are taken into account, highlighting the importance of phylogenetic tools for analyzing species pools not resulting from a random selection of taxa. Hardiness is the main factor explaining naturalization in Québec. Invasion requires dispersal, as shown by three significant variables associated with the spread of diaspores in the invasiveness model (seed weight, hydrochory, number of seed dispersal modes). Plants that are not cold‐hardy are likely to disappear from the market or nature, but the disappearance phenomenon is more complex, involving also seed dispersal abilities and propagule pressure. Factors contributing to naturalization or invasiveness may differ greatly between regions. Differences are due in part to the plant traits used in the models and the methodology. However, this study, conducted in a cold‐temperate region, sheds new light on what is likely a context (climatic)‐dependant phenomenon.

## 1 Introduction

Exotic and invasive vascular plant species are major threats to biodiversity and agricultural productivity. Only a fraction of the species accidentally or deliberately introduced naturalize, that is, survive and reproduce in nature without cultivation or ongoing introductions, and only a subset of these become invasive (spread over large distances and forms huge populations) or weedy (has detectable negative economic or environmental impacts; see Richardson, Pyšek, & Carlton, 2011 for definitions). However, the proportions (introduced vs. naturalized vs. invasive or weedy) greatly differ between regions (Richardson & Pyšek, 2012) and species groups (Pemberton & Liu, 2009). For instance, in Australia and New Zealand, the proportion of introduced species that naturalized varied from 9% in some families to 76% in others (Diez et al., 2009). In Britain, 68% of the species sold in nurseries from 1885 to 1985 escaped from cultivation (Dehnen‐Schmutz, Touza, Perrings, & Williamson, 2007). In Ireland, 48% of the exotic plants found in nature after 1970 have well‐established populations, and 19% are truly invasive (Milbau & Stout, 2008). Of the 1112 exotic species introduced (accidentally or deliberately) in the continental part of the United States and classified as invasive, 36% are considered noxious weeds (Lehan, Murphy, Thorburn, & Bradley, 2013). At the other end of the spectrum, only 10% of the 887 exotic species naturalized in Québec are weeds (Lavoie, Guay, & Joerin, 2014). In Hawaii, 5% of the 7866 ornamental species cultivated between 1840 and 1999 naturalized, and <1% became weeds (Schmidt & Drake, 2011).

These statistics indicate that predicting how many and which species will naturalize and eventually become invasive or weedy is an extremely difficult and context‐dependent task. Consequently, there is an urgent need to better understand the interactions between plant attributes and the processes which facilitate naturalization and invasiveness, to reduce uncertainties associated with predictions. This information will help plant biologists to develop efficient tools that can be used by environmental managers to prevent detrimental invasions. An ideal tool would focus on potential invaders, rather than species that only risk of becoming casual (do not form self‐replacing populations) or locally naturalized (Dehnen‐Schmutz, 2011; Milbau & Stout, 2008; Schmidt & Drake, 2011). In this respect, “failed” invasions can also be highly instructive for developing risk assessment support systems (Diez et al., 2009; Mack, 1991; Zenni & Nuñez, 2013).

The horticultural industry is a major player in the world plant market, with sales of about USD 109 billion in 2011 (Gyan Research and Analytics 2012). This industry is largely responsible for the introduction of exotic species in new regions or continents (Mack & Erneberg, 2002; Reichard & White, 2001). For instance, of the 671 invasive plants deliberately introduced in the continental United States, 426 (64%) were imported for ornamental purposes (Lehan et al., 2013). A large proportion of these species were introduced in the 19th century and in the first half of the 20th century (Lavoie, Saint‐Louis, Guay, Groeneveld, & Villeneuve, 2012; Mack, 1991), but the emergence of new horticultural trading partners from tropical regions, the Middle East, and Eastern Europe could be responsible for a new wave of plant invasions, underscoring the need for efficient risk assessment tools (Bradley et al., 2012).

Nursery catalogs can be extremely useful for identifying the characteristics of plants likely to naturalize or to become invaders (Dehnen‐Schmutz et al., 2007; Pemberton & Liu, 2009). They offer an excellent record of plants sold (although not necessarily bought by customers), and by comparing a list of catalog species with a list of naturalized species, those that escaped from gardens (successful naturalizations) can be easily distinguished from those that did not (“failed” invasions). Old (>100 years) catalogs are especially relevant for building models explaining naturalization, since the species sold for more than a century and that are still not found in nature are unlikely to naturalize in the future, at least under the present‐day climate.

The main objective of this study was to identify plant attributes associated with naturalization and invasiveness using century‐old nursery catalogs. This is not the first study of this kind (although there are only a few: Dehnen‐Schmutz et al., 2007; Pemberton & Liu, 2009; Skou, Pauleit, & Kollmann, 2012), and attempts to link invasiveness with plant attributes are multiplying (for reviews and debates on their relevance, see Pyšek & Richardson, 2007; van Kleunen, Weber, & Fischer, 2010; van Kleunen, Dawson, & Dostal, 2011; Thompson & Davis, 2011; Leffler, James, Monaco, & Sheley, 2014). However, we propose a new approach that takes into account not only plant attributes as explanatory factors, but also propagule pressure, while accounting for the nonindependence of the species analyzed due to their phylogenetic relationships. Museum collections were used, in combination with scientific journal databases, to assess invasiveness. We paid a particular attention to the species that never escaped from gardens and were thus potential cases of “failed” invasions. We tested the hypothesis of Richardson and Pyšek (2012) that naturalization is determined by fewer factors than invasion, as the latter also requires dispersal, which introduces additional complexity.

## 2 Methods

### 2.1 Taxon selection

This study was conducted using the ten nursery catalogs that were published in the province of Québec (Canada) in the 19th century, from 1817 to 1894, and that were still available from library archives (see Lavoie et al. (2012) for the complete list). The list of taxa sold in each catalog was first extracted. There were significant changes in nomenclature (in Latin, English, and/or French) over the last 200 years. Only taxa, including species, subspecies, varieties, and hybrids, for which the identification was certain, were retained. The taxonomic nomenclature was standardized using the Canadian Biodiversity Information Facility (2015) or Tropicos (Missouri Botanical Garden 2015) for taxa not listed in the former database.

Plants unable to escape from cultivation, that is, indoor taxa from tropical or equatorial regions (often listed as “greenhouse plants”), and taxa sold exclusively for human food production (fruits, vegetables) and with no ornamental value, were eliminated. The taxa were identified using various sources, such as ornamental plant guides, nursery catalogs, and agricultural or horticultural Web sites. The remaining taxa were mostly ornamental plants, but several were also probably sold for other purposes (e.g., medicinal plants).

We then identified the taxa no longer sold (in 2015) in Québec. Four sources of plant lists were used for identification: (i) Online catalogs from the four main nurseries of the province, including the biggest producers of annuals and perennials (Noël Wilson & Fils, Norseco, Pépinière Charlevoix, W.H. Perron), (ii) catalogs of custom horticultural tags (horticolor)—tags in French provide an indication of plants sold in the province, as they are exclusively produced for the Québec market, (iii) the search engine tool of the Association québécoise des producteurs en pépinière du Québec indicating which nurseries in the province produce a particular ornamental taxa, and (iv) the updated list of all trees available in Québec nurseries (Dumont, 2014, 2015). Taxa not found in at least one of the different plant lists were checked by two professional horticulturists cumulating 55 years of experience for detecting other taxa that were available to customers in 2015.

### 2.2 Naturalization and invasiveness characterization

The taxa from the catalogs that have naturalized were identified. The recent checklist of naturalized plants of Québec, published by Lavoie, Saint‐Louis, Guay, and Groeneveld (2012); updated in Lavoie et al., 2014), was used for this purpose. Finally, we identified which naturalized taxa had become invasive. No plant atlas was available for Québec, so to identify the invasive taxa, the number of herbarium specimens stored in the two main herbaria of the province, MT (Université de Montréal) and QFA (Université Laval), was used as a surrogate measure of the number of occurrences in Québec (see Lavoie, Shah, Bergeron, & Villeneuve, 2013; for methodological details). MT and QFA harbor about 80% of the 1,800,000 vascular plant specimens stored in Québec herbaria (Thiers, 2016). In general, the number of specimens is a good indicator of the size of a plant population in the field (MacDougall, Loo, Clayden, Goltz, & Hinds, 1998; Phillips, Brown, Dixon, & Hopper, 2011; Puyravaud, Davidar, Pascal, & Ramesh, 2003; Vetaas, 2000; Wu, Rejmánek, Grotkopp, & DiTomaso, 2005). However, common (~invasive) and rare (~noninvasive) species are usually under‐ or over‐represented in herbaria (Garcillán & Ezcurra, 2011; Garcillán, Ezcurra, & Vega, 2008), which can potentially reduce the statistical power of analyses conducted with specimen data.

Preliminary tests using only the number of specimens as dependant variable in multiple regression models had a poor performance for explaining invasiveness. This performance was likely related to the under‐representation of species that spread mainly during the last 30–40 years, a period with a very low specimen collection effort in Québec (Lavoie et al., 2012). We nevertheless estimated that the number of specimens was a reliable source of data, as long as it was combined with another indicator of invasiveness, the scientific research effort. This effort, estimated using the number of published scientific papers, provides an indirect measurement of invasiveness: The more invasive the species, the more it attracts the attention of scientists, and the more papers focussing on this species are likely to be published (Lavoie et al., 2014). The scientific research effort was estimated using the Web of Science^™^ database (Thomson Reuters 2013; last query: 10 December 2013) with the name of the taxa (in Latin) and the keyword “invasive” or “invasion” in the “title” or “topic” research fields, to extract the associated papers. Each paper was checked for relevance. Only studies clearly related to the taxa of interest and conducted in northeastern North America (the area covered by the flora of Gleason & Cronquist, 1991), that is, in a region roughly similar to Québec from a climatic and vegetation point of view, were retained. Data on the number of specimens and the number of papers were first cubic‐root transformed to normalize their distribution and then standardized on a 0–1 scale to give equal weight to the variables before analysis. On these two sets of variables, a k‐mean clustering algorithm (iterated 100 times) was run in R software (R Development Core Team 2013) to partition the naturalized taxa group into *k* = 2 subgroups (invasive or noninvasive).

### 2.3 Plant attributes and propagule pressure

A database of plant attributes was generated for the taxa listed in the catalogs. Retained attributes were those readily accessible from online databases or the scientific literature and available for all taxa (Table 1). For perennials, the hardiness zone variables were derived using the methodology developed by Lavoie et al. (2013), essentially based on the overlap between the geographic distribution of the taxa in the native and exotic ranges and hardiness zone maps. For annuals, hardiness zones are less relevant to horticulturists. However, several ornamental plant guides and Web sites provide information on the lowest temperature a taxon can tolerate, which was used to estimate the coldest hardiness zone; warmer zones were assumed to be tolerated by the taxon. A similar approach has been successfully used in the past to compare the invasion probability of annuals and perennials from historical catalogs (Dehnen‐Schmutz et al., 2007).

**Table 1:** Plant attributes that were used to explain the naturalization, invasiveness, and disappearance of taxa listed in nursery catalogs published in the 19th century in Québec (Canada)

Plant attribute	Description	Variable type	Main sources
1. Life cycle	Annual	Binary (0/1)	United States Department of Agriculture (2015a)
2. Woody	With woody tissues	Binary (0/1)	United States Department of Agriculture (2015a)
3. Vine	With a stem that climbs by winding itself on a support	Binary (0/1)	United States Department of Agriculture (2015a)
3. Plant height	Maximum height (cm)	Continuous	Fitter and Peat (1994); Marie‐Victorin (1995); Rice (2006)
4. Seed weight	Weight of 1000 seeds (g)	Continuous	Kleyer et al. (2008); Royal Botanic Gardens Kew (2015)
5. Main seed dispersal mode	5a. Anemochory	Binary (0/1)	Fitter and Peat (1994); Julve (1998); Royal Botanic Gardens Kew (2015)
	5b. Autochory	Binary (0/1)	
	5c. Hydrochory	Binary (0/1)	
	5d. Zoochory (epi or endo)	Binary (0/1)	
	5e. Number of modes (main and secondary)	Discrete (1–4)	
6. Vegetative reproduction	Able to reproduce vegetatively	Binary (0/1)	Fitter and Peat (1994); Klimešová and Klimeš (2015); United States Department of Agriculture (2015a)
7. Native range region	7a. Africa	Binary (0/1)	United States Department of Agriculture (2015b)
	7b. Asia (temperate area)	Binary (0/1)	
	7c. Asia (tropical area)	Binary (0/1)	
	7d. Europe	Binary (0/1)	
	7e. North America	Binary (0/1)	
	7f. South America	Binary (0/1)	
	7g. Number of native range regions	Discrete (1–6)	
8. Hardiness	8a. Number of hardiness zones covered by the plant	Discrete (1–11)	See Lavoie et al. (2013) for methodological details and main sources (especially Natural Resources Canada, 2015, and United States Department of Agriculture, 2015c); various ornamental plant guides and websites
	8b. Coldest hardiness zone tolerated by the plant	Discrete (1–11); the colder the zone, the lower the number	

Two variables were used as indicators of propagule pressure (sensu Lockwood, Cassey, & Blackburn, 2005), that is, the number of catalogs in which the taxon was listed and the number of years elapsed since its first mention in a catalog. We hypothesized that a taxon available from more nurseries and sold for a longer period of time would be more widely planted, thus producing more propagules with the potential to escape from gardens and to contribute to naturalization and/or invasiveness (Dehnen‐Schmutz et al., 2007; Pemberton & Liu, 2009; Pyšek, Křivánek, & Jarošík, 2009; Skou et al., 2012).

### 2.4 Statistical models and phylogenies

Three logistic regression models (Hosmer & Lemeshow, 2000) were constructed for this study. The naturalization (or not) of a taxon included in at least one of the ornamental plant catalogs was the dependent variable of the first model (the *naturalization* model). Whether a taxon from the catalogs became invasive (or not) was the dependent variable of the second model (the *invasiveness* model). That a taxon from the catalogs was neither sold nor naturalized in 2015 (or still sold and/or naturalized) was the dependent variable of the third model (the *disappearance* model, in that the taxon was no longer found in Québec in nurseries or in nature, albeit potentially still present in gardens). The remaining variables (plant attributes, propagule pressure) were integrated in a first series of models as independent (or explanatory) variables. Prior to including plant attributes in the analyses, categorical data were coded into binary dummy variables, continuous data were log‐transformed to normalize their distribution, and discrete and continuous data were standardized by subtracting the mean of each variable and dividing by two times its standard deviation to facilitate comparisons with the dummy variables (see Gelman, 2008 for details). Linearly dependent variables and variables that showed high collinearity (VIF > 3) in the full models were removed. A forward stepwise model selection was then performed to construct the logistic regression models and finally select the best models based on the Akaike information criterion (AIC; Burnham & Anderson, 2002). All models were run in R software (R Development Core Team 2013).

Plants found in catalogs are by no means a random selection of species. To verify whether the models elaborated in this study were taxonomically or phylogenetically biased, logistic regressions correcting for phylogenetic correlations in the residuals of the models (Ives & Garland, 2010) were also performed (hereafter named phylogenetic logistic regressions). Phylogenies for the taxa were obtained from the online tool phylomatic version 3 (Webb & Donoghue, 2005), which is based on the APG III classification system (Angiosperm Phylogeny Group 2009). Node ages were calibrated with data from Wikström, Savolainen, and Chase (2001), and branch lengths were adjusted using the bladj tool in phylocom (Webb, Ackerly, & Kembel, 2008). The R package *phylolm* (Ho & Ane, 2014) was then used to run a second series of logistic regression models with the whole phylogeny included as a covariance structure. This approach assumes that the residual variation follows a homogeneous model of evolution across the branches of the phylogenetic tree, and a violation of this assumption could lead to unacceptable type I error rates and/or reduced statistical power (Mazel et al., 2016). For each model, this assumption was tested by looking for rate shifts in the residuals of a standard logistic regression along the phylogeny using the *auteur* approach (Eastman, Alfaro, Joyce, Hipp, & Harmon, 2011) from the *geiger* package in R, as recommended by Mazel et al. (2016). Rate shifts were detected in all models, but the vast majority (naturalization), or almost all (invasiveness) if not all (disappearance), of these shifts occurred on branches sustaining either one species or one genera. Consequently, we concluded that the rate shifts did not have phylogenetic structure in the residuals and decided to perform the regressions with the unmodified phylogeny. As for the nonphylogenetic models, a forward stepwise selection procedure based on the AIC was used to select the best models. These models, with and without phylogeny, were analyzed side by side‐to‐see how incorporating phylogeny affected the significance of species attributes directly. McFadden's pseudo *R* ^2^, correcting for the number of parameters included in the model ($R_{\text{adj}}^{2}$), was estimated for each model.

## 3 Results

**Figure 1:**
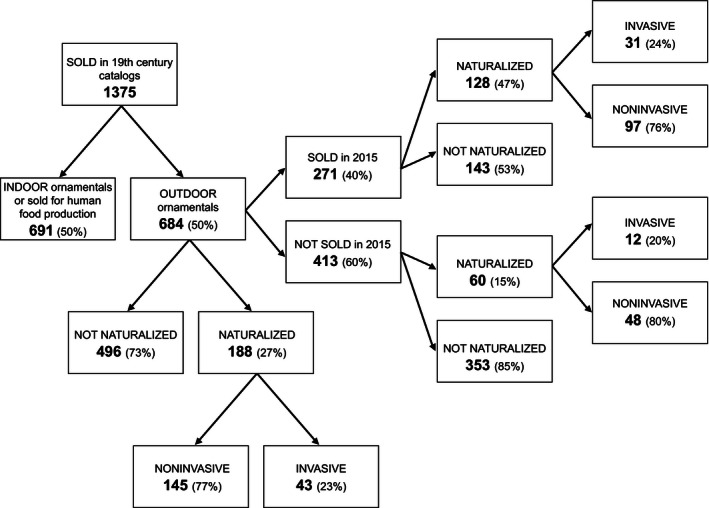
Number of plant taxa listed in nursery catalogs published in Québec (Canada) in the 19th century, classified according to their status

A total of 1375 plant taxa were listed in the nursery catalogs published in Québec in the 19th century (Fig. 1). However, only 684 taxa (668 different species, two species with two subspecies, respectively, and 14 hybrids), grouped into 98 families, were truly ornamental outdoor plants (Table S1). Seven families (Asteraceae, Caryophyllaceae, Fabaceae, Iridaceae, Lamiaceae, Ranunculaceae, and Rosaceae) represented 37% of the 684 taxa. The genera with the highest number of taxa were *Iris* (18), *Rosa* (17), *Silene* (11), *Primula* (10), *Clematis* (9), and *Lilium* (9). About 24% of the taxa were annuals and 28% woody plants. They were essentially introduced from Europe (44%), temperate Asia (44%), North America (29%), and Africa (20%)—the native range often spanned more than one continent.

Among the 684 outdoor taxa, 188 (27%), representing 61 families, naturalized (Fig. 1; Table S1). None of the 14 hybrids naturalized. Asteraceae, Caryophyllaceae, Fabaceae, Lamiaceae, Ranunculaceae, and Rosaceae were still well represented (35% of the taxa), but only four of the 20 Iridaceae taxa naturalized. No genera had more than six taxa. Among the naturalized taxa, 22% were annuals and 26% woody plants. Most were introduced from Europe (66%), temperate Asia (59%), North America (24%), and Africa (21%). The k‐mean clustering algorithm partitioned the group of naturalized taxa into two subgroups (Table S1): one containing 43 invasives (23%) and the other 145 noninvasives (77%). The invasive and noninvasive subgroups had a mean number of herbarium specimens of 252 and 13, and a mean number of scientific papers of 7.0 and 0.4, respectively.

About 40% (271 of 684) of the taxa sold in the 19th century were still available on the Québec market in 2015 (Fig. 1; Table S1). Of them, 128 naturalized (47%), and among the naturalized taxa, 31 (24%) became invasive. By contrast, among the taxa no longer sold (413), only 60 (15%) naturalized, and 12 of them (20%) became invasive. Again, Asteraceae, Caryophyllaceae, Fabaceae, Lamiaceae, Ranunculaceae, and Rosaceae were still well represented in both groups (sold: 36%; not sold: 32%), but another group of families (Boraginaceae, Brassicaceae, Iridaceae, Liliaceae, Poaceae, Scrophulariaceae, and Solanaceae) was clearly more represented in the no longer sold group (23%) than in the still sold group (11%). These groups also differed on other aspects (Table 2), but these differences varied according to the status of the plant (naturalized or not).

**Table 2:** Attributes (mean or median values, proportion of taxa) characterizing plant taxa listed in nursery catalogs published in the 19th century in Québec (Canada), according to whether the taxa were still sold (or not) in the province in 2015, and according to their status (naturalized or not)

Plant attribute	Sold in 2015Naturalized	Not naturalized	Not sold in 2015 Naturalized	Not naturalized
*n* taxa	128	143	60	353
Annual (% of taxa)	16.4	14.0	35.0	29.5
Woody (% of taxa)	32.0	36.4	13.3	26.3
Maximum plant height (median value; cm)	120	100	60	90
Seed weight (1000 seeds; median value; g)	3.0	5.1	1.6	3.0
Main seed dispersal mode				
Anemochory (% of taxa)	22.7	25.9	26.7	22.1
Autochory (% of taxa)	27.3	37.8	35.0	45.6
Hydrochory (% of taxa)	7.0	4.2	1.7	5.7
Zoochory (% of taxa)	43.0	32.2	36.7	26.6
Vegetative reproduction (% of taxa)	60.2	69.9	48.3	49.6
Native range region				
Africa (% of taxa)	18.0	14.0	28.3	22.7
Asia (temperate area; % of taxa)	58.6	44.8	60.0	35.4
Asia (tropical area; % of taxa)	9.4	7.7	8.3	7.6
Europe (% of taxa)	60.9	38.5	76.7	34.0
North America (% of taxa)	27.3	31.5	18.3	30.9
South America (% of taxa)	5.5	7.7	1.7	13.9

The logistic regression models (Table 3) had $R_{\text{adj}}^{2}$ ranging from 0.248 to 0.292. Phylogeny affected the significance of plant attribute or propagule pressure variables only in the naturalization model, by reducing the number of significant variables from ten to two: A taxon sold in the 19th century in Québec was more likely to naturalize if it was cold‐hardy and did not tolerate a wide range of hardiness zones. Some examples are *Campanula trachelium* (Campanulaceae), *Geranium pratense* (Geraniaceae), and *Silene chalcedonica* (Caryophyllaceae).

**Table 3:** Standard logistic regression and phylogenetic logistic regression models explaining the naturalization, invasiveness, and disappearance (plants neither sold nor naturalized in 2015) of plant taxa listed in nursery catalogs published in the 19th century in Québec (Canada). Only significant variables are shown

Standard logistic regression					Phylogenetic logistic regression				
Model and explanatory variable	Estimate	Standard error	*z* value	*p*	Model and explanatory variable	Estimate	Standard error	*z* value	*p*
Explaining naturalization ($R_{\mathrm{adj}}^{2}$ = .282)					Explaining naturalization ($R_{\mathrm{adj}}^{2}$ = .248)				
Annual	1.030	0.284	3.621	.0003***					
Seed weight	–0.467	0.221	–2.116	.0343*					
Main seed dispersal mode: autochory	–0.812	0.256	–3.176	.0015**					
Number of seed dispersal modes	0.817	0.204	4.000	<.0001***					
Native range: Asia (temperate)	0.622	0.273	2.280	.0226*					
Native range: Europe	1.021	0.284	3.597	.0003***					
Native range: North America	1.040	0.372	2.795	.0052**					
Number of hardiness zones covered by the species	–2.465	0.338	–7.300	<.0001***	Number of hardiness zones covered by the species	–1.039	0.369	–2.817	<.0049**
Coldest hardiness zone tolerated by the species	–4.402	0.483	–9.120	<.0001***	Coldest hardiness zone tolerated by the species	–1.774	0.585	–3.030	<.0024**
Number of catalogs	0.897	0.205	4.375	<.0001***					
Intercept	–2.505	0.384	–6.517	<.0001***	Intercept	2.981	0.776	3.841	.0001***
Explaining invasiveness ($R_{\mathrm{adj}}^{2}$_ _= 0.283)					Explaining invasiveness ($R_{\mathrm{adj}}^{2}$_ _= 0.287)				
Vine	1.281	0.614	2.085	.0371*	Vine	1.174	0.387	3.036	.0024**
Seed weight	–1.008	0.429	–2.350	.0188*	Seed weight	–0.489	0.241	–2.032	.0421*
Main seed dispersal mode: hydrochory	1.536	0.671	2.290	.0220*	Main seed dispersal mode: hydrochory	0.844	0.399	2.115	.0344*
Number of seed dispersal modes	0.759	0.285	2.658	.0079**	Number of seed dispersal modes	0.491	0.179	2.748	.0060**
Native range: Asia (temperate)	1.162	0.437	2.660	.0078**	Native range: Asia (temperate)	0.787	0.224	3.512	.0004***
Native range: Europe	1.336	0.472	2.827	.0047**	Native range: Europe	0.926	0.245	3.786	.0002***
Coldest hardiness zone tolerated by the species	–5.001	0.869	–5.756	<.0001***	Coldest hardiness zone tolerated by the species	–2.614	0.530	–4.934	<.0001***
Intercept	–6.443	0.730	–8.823	<.0001***	Intercept	–3.746	0.850	–4.404	<.0001***
Explaining disappearance ($R_{\mathrm{adj}}^{2}$ ^ ^= .290)					Explaining disappearance ($R_{\mathrm{adj}}^{2}$ ^ ^= .292)				
Main seed dispersal mode: autochory	0.547	0.198	2.762	.0058**	Main seed dispersal mode: autochory	0.538	0.203	2.657	.0079**
Number of seed dispersal modes	–0.517	0.198	–2.613	<.0090**	Number of seed dispersal modes	–0.559	0.199	–2.812	<.0049**
Number of hardiness zones covered by the species	1.691	0.273	6.183	.0001***	Number of hardiness zones covered by the species	1.631	0.276	5.899	<.0001***
Coldest hardiness zone tolerated by the species	4.077	0.359	11.361	<.0001***	Coldest hardiness zone tolerated by the species	3.932	0.360	10.917	<.0001***
Number of catalogs	–1.431	0.237	–6.038	<.0001***	Number of catalogs	–1.403	0.236	–5.956	<.0001***
Intercept	–0.018	0.126	–0.145	.8851	Intercept	0.013	0.166	0.767	.4432

*p <* *.05; **p <* *.01; ***p <* *.001.

Invasiveness was explained by seven variables (Table 3). In summary, a taxon sold in the 19th century in Québec was more likely to become invasive if it was a vine with light seeds, had several dispersal modes (especially by water), was introduced from temperate Asia or Europe, and was cold‐hardy. Few taxa have, of course, all these characteristics, but examples of invasive plants sharing most of these attributes are *Lythrum salicaria* (Lythraceae), *Myosotis scorpioides* (Boraginaceae), and *Solanum dulcamara* (Solanaceae).

**Figure 2:**
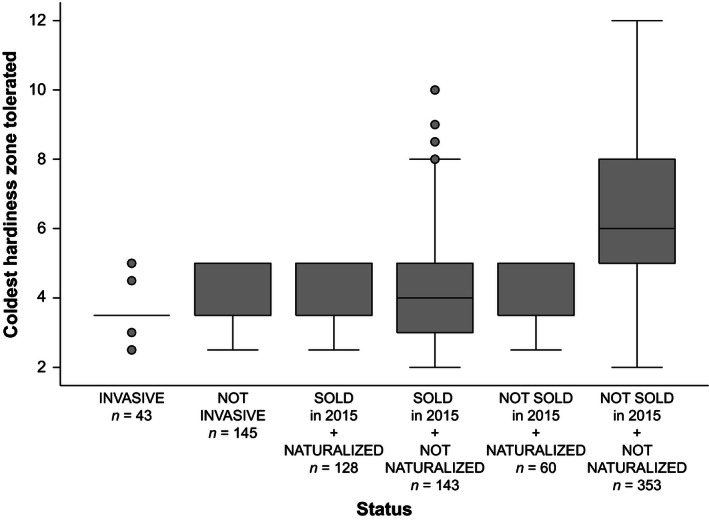
Boxplots illustrating the coldest hardiness zone tolerated by plant taxa listed in nursery catalogs published in Québec (Canada) in the 19th century, classified according to their status

The disappearance was explained by five variables (Table 3). A taxon from the list of plants sold in the 19th century was more likely to “disappear” from Québec (no naturalization, no longer sold) if it had only a small number of seed dispersal modes, especially if its main mode was autochory, was not cold‐hardy (Fig. 2) but nevertheless tolerated a wide range of hardiness zones, and was found in only a few catalogs. Some examples are *Glandularia platensis* (Verbenaceae, from Argentina, Brazil, Paraguay, and Uruguay; sold in 1878 and 1881), *Lupinus tomentosus* (Fabaceae, from Bolivia and Peru; sold in 1834), and *Phacelia viscida* (Boraginaceae, from Mexico and California; sold in 1878 and 1881).

## 4 Discussion

The models constructed with the historical nursery catalogs published in Québec show that naturalization in cold‐temperate environments is determined by fewer factors than invasion. However, this conclusion was reached only when phylogenetic relationships were taken into account, highlighting the importance of phylogenetic tools for analyzing species pools not resulting from a random selection of taxa. This is especially true for plant catalogs, given the strong preference of horticulturists for certain families and genera with high ornamental value (e.g., *Iris*,* Rosa*,* Primula*, and *Lilium*).

Hardiness is the main factor explaining naturalization in Québec; plants tolerating a wider range of hardiness zones are also less likely to naturalize, but regardless of the number of zones, if a plant is not cold‐hardy, its establishment and survival chances are low. In a cold‐temperate region such as Québec, cold hardiness as an explanatory variable is unsurprising, but that hardiness is the only significant attribute for naturalization is especially revealing. Cold hardiness is not a plant trait by itself: It is an indicator of a combination of morphological and physiological traits allowing plants to survive cold temperatures, and especially frost (United States Department of Agriculture 2015c). In Québec, being cold‐frost resistant is necessary for naturalization and for the transition from naturalization to invasiveness, but other attributes not included in our models probably help the establishment and expansion of populations over large areas, such as a long flowering time, a large specific leaf area, and the presence of adequate pollinators (Bufford & Daehler, 2014; Gallagher, Randall, & Leishman, 2014). The importance of cold tolerance is highlighted by the analysis of ornamental plants that never naturalized and are no longer sold in Québec, which, as a group, are much less cold‐hardy than the other plants. Nurseries and horticulturists of the 19th century probably selected outdoor plants by trial and error, and species that were not cold tolerant enough were rapidly discarded because they were not well adapted to the short growing season and cold winters in Québec. On the other hand, the fact that plants now naturalized were first nurtured in gardens probably facilitated the establishment of viable populations, by buffering against the first critical filters of the introduction–naturalization–invasion continuum associated with environmental stochasticity and propagule pressure (Mack et al., 2000; Moodley, Geerts, Rebelo, Richardson, & Wilson, 2014; Richardson & Pyšek, 2012).

Richardson and Pyšek (2012) proposed that invasion requires dispersal and this is effectively shown by the three significant variables associated with the spread of diaspores in the invasiveness model (seed weight, hydrochory, number of seed dispersal modes). Temperate Asia (particularly eastern Asia) and Europe are major donors of naturalized plants to other continents, and especially to North America (Guo, Qian, Ricklefs, & Xi, 2006; van Kleunen et al., 2015; Rejmánek, 2014; Stohlgren et al., 2011), so it is not surprising to see European and Asian origin as significant explanatory variables in the invasiveness model. Plants from Asia (temperate) and/or Europe form 60% of the catalog taxa pool, but 86% of the invasive species identified in this study.

Plants that are not sufficiently cold‐hardy are likely to “disappear” from the market or nature in Québec, but the disappearance phenomenon is more complex, involving low or short‐distance seed dispersal abilities (e.g., autochory) and low propagule pressure (not widely sold). To our knowledge, this study is the first to analyze the disappearance phenomenon from a large pool of ornamental plants. It is noteworthy that propagule pressure—as estimated from plant sale data—is often identified as a an important, if not the most important, determinant of naturalization for ornamental or cultivated plants (Dehnen‐Schmutz et al., 2007; Moodley et al., 2014; Pemberton & Liu, 2009; Pyšek et al., 2009, 2009; Skou et al., 2012). However, in a cold region such as Québec, not being sold (low propagule pressure) is at least partially dependant on a lack of cold hardiness.

Factors contributing to naturalization or invasiveness may differ greatly between regions, as indicated by similar studies conducted in Australia, Central Europe, Hawaii, or Ireland (Gallagher et al., 2014; Milbau & Stout, 2008; Moodley, Geerts, Richardson, & Wilson, 2013; Pyšek et al., 2009; Schmidt & Drake, 2011). Differences rely in part on plant traits used in the models—some are almost always used (e.g., maximum height, seed mass), others rarely (specific leaf area), some included phylogeny, others not, etc. However, this study, conducted in a cold‐temperate region, sheds new light on what is likely a context (climatic)‐dependant phenomenon.

Using the naturalization model constructed in this study as a tool to predict the naturalization of a newly introduced plant would be risky, because only about a quarter of the variation was explained. The invasiveness model—the most important from an environmental management perspective—is of limited use for the industry, as nurseries in Québec do not typically sell outdoor plants that are not, for instance, cold‐hardy. On the other hand, it highlights the challenge this industry will face in an ever warming world: Hardiness zones are likely to shift northward over the next decades (Bradley et al., 2012), and several species currently sold could soon transition from casual to naturalized to invasive, causing additional pressure on native plant diversity. Regularly updating the cold hardiness zone maps would help rapidly flag new potential invaders, and banning the sale of invasive and weedy species in Québec—a list of such species has recently been compiled (Lavoie et al., 2014)—could be part of a solution. Unfortunately, there is actually no political will in the province to tackle this problem.

## Funding information

This research was financially supported by the Natural Sciences and Engineering Research Council of Canada (194613‐2013), the Fédération Interdisciplinaire de l'horticulture Ornementale du Québec, Université Laval, and the Institut Hydro‐Québec Environnement, Développement et Société.

## Conflict of interest

None declared.

## References

[ece32471-bib-0001] Angiosperm Phylogeny Group (2009). An update of the Angiosperm Phylogeny Group classification for the orders and families of flowering plants: APG III. Botanical Journal of the Linnean Society, 161, 105–121.

[ece32471-bib-0002] Bradley, B. A. , Blumenthal, D. M. , Early, R. , Grosholz, E. D. , Lawler, J. J. , Miller, L. P. , … Olden, J. D. (2012). Global change, global trade, and the next wave of invasions. Frontiers in Ecology and the Environment, 10, 20–28.

[ece32471-bib-0003] Bufford, J. L. , & Daehler, C. C. (2014). Sterility and lack of pollinator services explain reproductive failure in non‐invasive ornamental plants. Diversity and Distributions, 20, 975–985.

[ece32471-bib-0004] Burnham, K. P. , & Anderson, D. R. (2002). Model selection and multi‐model inference: A practical information – theoretic approach. Fort Collins, CO: Springer.

[ece32471-bib-0005] Canadian Biodiversity Information Facility (2015). Integrated taxonomic information system. Retrieved from http://www.cbif.gc.ca/pls/itisca/taxaget?p_ifx=cbif.

[ece32471-bib-0006] Dehnen‐Schmutz, K. (2011). Determining non‐invasiveness in ornamental plants to build green lists. Journal of Applied Ecology, 48, 1374–1380.

[ece32471-bib-0007] Dehnen‐Schmutz, K. , Touza, J. , Perrings, C. , & Williamson, M. (2007). A century of the ornamental plant trade and its impact on invasion success. Diversity and Distributions, 13, 527–534.

[ece32471-bib-0008] Diez, J. M. , Williams, P. A. , Randall, R. P. , Sullivan, J. J. , Hulme, P. E. , & Duncan, R. P. (2009). Learning from failures: Testing broad taxonomic hypotheses about plant naturalization. Ecology Letters, 12, 1174–1183. 19723283 10.1111/j.1461-0248.2009.01376.x

[ece32471-bib-0009] Dumont, B. (2014). Arbres pour les municipalités du Québec et l'est de l'Ontario. Tome II. Boucherville, QC: Horti Média.

[ece32471-bib-0010] Dumont, B. (2015). Des arbres pour les jardins paysagers. Québec City, QC: MultiMondes.

[ece32471-bib-0011] Eastman, J. M. , Alfaro, M. E. , Joyce, P. , Hipp, A. L. , & Harmon, L. J. (2011). A novel comparative method for identifying shifts in the rate of character evolution on trees. Evolution, 65, 3578–3589. 22133227 10.1111/j.1558-5646.2011.01401.x

[ece32471-bib-0012] Fitter, A. H. , & Peat, H. J. (1994). The Ecological Flora Database. Journal of Ecology, 82, 415–425.

[ece32471-bib-0013] Gallagher, R. V. , Randall, R. P. , & Leishman, M. R. (2014). Trait differences between naturalized and invasive plant species independent of residence time and phylogeny. Conservation Biology, 29, 360–369. 25369762 10.1111/cobi.12399 PMC4405095

[ece32471-bib-0014] Garcillán, P. P. , & Ezcurra, E. (2011). Sampling procedures and species estimation: Testing the effectiveness of herbarium data against vegetation sampling in an oceanic island. Journal of Vegetation Science, 22, 273–280.

[ece32471-bib-0015] Garcillán, P. P. , Ezcurra, E. , & Vega, E. (2008). Guadalupe Island: Lost paradise recovered? Overgrazing impact on extinction in a remote oceanic island as estimated through accumulation functions. Biodiversity and Conservation, 17, 1613–1625.

[ece32471-bib-0016] Gelman, A. (2008). Scaling regression inputs by dividing by two standard deviations. Statistics in Medicine, 27, 2865–2873. 17960576 10.1002/sim.3107

[ece32471-bib-0017] Gleason, H. A. , & Cronquist, A. (1991). Vascular plants of northeastern United States and adjacent Canada. New York, NY: New York Botanical Garden.

[ece32471-bib-0018] Guo, Q. , Qian, H. , Ricklefs, R. E. , & Xi, W. (2006). Distributions of exotic plants in eastern Asia and North America. Ecology Letters, 9, 827–834. 16796573 10.1111/j.1461-0248.2006.00938.x

[ece32471-bib-0019] Gyan Research and Analytics (2012). Global horticulture market outlook 2015. New Delhi, India: Gyan Research and Analytics.

[ece32471-bib-0020] Ho, L. S. T. , & Ane, C. (2014). A linear‐time algorithm for Gaussian and non‐Gaussian trait evolution models. Systematic Biology, 63, 397–408. 24500037 10.1093/sysbio/syu005

[ece32471-bib-0021] Hosmer, D. W. , & Lemeshow, S. (2000). Applied logistic regression. New York, NY: John Wiley and Sons.

[ece32471-bib-0022] Ives, A. R. , & Garland, T. Jr (2010). Phylogenetic logistic regression for binary dependent variables. Systematic Biology, 59, 9–26. 20525617 10.1093/sysbio/syp074

[ece32471-bib-0023] Julve, P. (1998). Baseflor: index botanique, écologique et chorologique de la flore de France. Retrieved from http://philippe.julve.pagesperso-orange.fr/catminat.htm#INDEXFLORE.

[ece32471-bib-0024] Kleyer, M. , Bekker, R. M. , Knevel, I. C. , Bakker, J. P. , Thompson, K. , Sonnenschein, M. , … Peco, B. (2008). The LEDA Traitbase: A database of life‐history traits of the Northwest European flora. Journal of Ecology, 96, 1266–1274.

[ece32471-bib-0025] Klimešová, J. , & Klimeš, L. (2015). Clo‐Pla3: database of clonal growth of plants from Central Europe. Retrieved from http://clopla.butbn.cas.cz.

[ece32471-bib-0026] Lavoie, C. , Guay, G. , & Joerin, F. (2014). Une liste des plantes vasculaires exotiques nuisibles du Québec: Nouvelle approche pour la sélection des espèces et l'aide à la décision. Écoscience, 21, 133–156.

[ece32471-bib-0027] Lavoie, C. , Saint‐Louis, A. , Guay, G. , & Groeneveld, E. (2012). Les plantes vasculaires exotiques naturalisées: Une nouvelle liste pour le Québec. Le Naturaliste Canadien, 136(3), 6–32.

[ece32471-bib-0028] Lavoie, C. , Saint‐Louis, A. , Guay, G. , Groeneveld, E. , & Villeneuve, P. (2012). Naturalization of exotic plant species in north‐eastern North America: Trends and detection capacity. Diversity and Distributions, 18, 180–190.

[ece32471-bib-0029] Lavoie, C. , Shah, M. A. , Bergeron, A. , & Villeneuve, P. (2013). Explaining invasiveness from the extent of native range: New insights from plant atlases and herbarium specimens. Diversity and Distributions, 19, 98–105.

[ece32471-bib-0030] Leffler, A. J. , James, J. J. , Monaco, T. A. , & Sheley, R. L. (2014). A new perspective on trait differences between native and invasive exotic plants. Ecology, 95, 298–305. 24669724 10.1890/13-0102.1

[ece32471-bib-0031] Lehan, N. E. , Murphy, J. R. , Thorburn, L. P. , & Bradley, B. A. (2013). Accidental introductions are an important source of invasive plants in the continental United States. American Journal of Botany, 100, 1287–1293. 23825135 10.3732/ajb.1300061

[ece32471-bib-0032] Lockwood, J. L. , Cassey, P. , & Blackburn, T. (2005). The role of propagule pressure in explaining species invasions. Trends in Ecology & Evolution, 20, 223–228. 16701373 10.1016/j.tree.2005.02.004

[ece32471-bib-0033] MacDougall, A. S. , Loo, J. A. , Clayden, S. R. , Goltz, J. G. , & Hinds, H. R. (1998). Defining conservation priorities for plant taxa in southeastern New Brunswick, Canada using herbarium records. Biological Conservation, 86, 325–338.

[ece32471-bib-0034] Mack, R. N. (1991). The commercial seed trade: An early disperser of weeds in the United States. Economic Botany, 45, 257–273.

[ece32471-bib-0035] Mack, R. N. , & Erneberg, M. (2002). The United States naturalized flora: Largely the product of deliberate introductions. Annals of the Missouri Botanical Garden, 89, 176–189.

[ece32471-bib-0036] Mack, R. N. , Simberloff, D. , Lonsdale, W. M. , Evans, H. , Clout, M. , & Bazzaz, F. A. (2000). Biotic invasions: Causes, epidemiology, global consequences, and control. Ecological Applications, 10, 689–710.

[ece32471-bib-0037] Marie‐Victorin, F. (1995). Flore laurentienne. Montréal, QC: Presses de l'Université de Montréal.

[ece32471-bib-0038] Mazel, F. , Davies, J. T. , Georges, D. , Lavergne, S. , Thuiller, W. , & Peres‐Neto, P. R. (2016). Improving phylogenetic regression under complex evolutionary models. Ecology, 97, 286–293. 27145604 10.1890/15-0086.1 PMC5486445

[ece32471-bib-0039] Milbau, A. , & Stout, J. C. (2008). Factors associated with alien plants transitioning from casual, to naturalized, to invasive. Conservation Biology, 22, 308–317. 18261149 10.1111/j.1523-1739.2007.00877.x

[ece32471-bib-0040] Missouri Botanical Garden . (2015). Tropicos. Retrieved from http://www.tropicos.org.

[ece32471-bib-0041] Moodley, D. , Geerts, S. , Rebelo, T. , Richardson, D. M. , & Wilson, J. R. U. (2014). Site‐specific conditions influence plant naturalization: The case of alien Proteaceae in South Africa. Acta Oecologica, 59, 62–71.

[ece32471-bib-0042] Moodley, D. , Geerts, S. , Richardson, D. M. , & Wilson, J. R. U. (2013). Different traits determine introduction, naturalization and invasion success in woody plants: proteaceae as a test case. PLoS ONE, 8, e75078. doi:doi.org/10.1371/journal.pone.0075078 24086442 10.1371/journal.pone.0075078 PMC3782508

[ece32471-bib-0043] Natural Resources Canada (2015) Plant hardiness of Canada. Retrieved from http://www.planthardiness.gc.ca.

[ece32471-bib-0044] Pemberton, R. W. , & Liu, H. (2009). Marketing time predicts naturalization of horticultural plants. Ecology, 90, 69–80. 19294914 10.1890/07-1516.1

[ece32471-bib-0045] Phillips, R. D. , Brown, A. P. , Dixon, K. W. , & Hopper, S. D. (2011). Orchid biogeography and factors associated with rarity in a biodiversity hotspot, the Southwest Australian Floristic Region. Journal of Biogeography, 38, 487–501.

[ece32471-bib-0046] Puyravaud, J.‐P. , Davidar, P. , Pascal, J.‐P. , & Ramesh, B. R. (2003). Analysis of threatened endemic trees of the Western Ghats of India sheds new light on the Red Data Book of Indian Plants. Biodiversity and Conservation, 12, 2091–2106.

[ece32471-bib-0047] Pyšek, P. , Jarošík, V. , Pergl, J. , Randall, R. , Chytrý, M. , Kühn, I. , … Sádlo, J. (2009). The global invasion success of Central European plants is related to distribution characteristics in their native range and species traits. Diversity and Distributions, 15, 891–903.

[ece32471-bib-0048] Pyšek, P. , Křivánek, M. , & Jarošík, V. (2009). Planting intensity, residence time, and species traits determine invasion success of alien woody species. Ecology, 90, 2734–2744. 19886483 10.1890/08-0857.1

[ece32471-bib-0049] Pyšek, P. , & Richardson, D. M. (2007). Traits associated with invasiveness in alien plants: Where do we stand? In Nentwig W. (Ed.), Biological invasions (pp. 97–125). Berlin, Germany: Springer.

[ece32471-bib-0050] R Development Core Team (2013). R: A language and environment for statistical computing. Vienna, Austria: R Foundation for Statistical Computing.

[ece32471-bib-0051] Reichard, S. H. , & White, P. (2001). Horticulture as a pathway of invasive plant introductions in the United States. BioScience, 51, 103–113.

[ece32471-bib-0052] Rejmánek, M. (2014). Invasive trees and shrubs: Where do they come from and what we should expect in the future? *Biological* Invasions, 16, 483–498.

[ece32471-bib-0053] Rice G. (Ed.) (2006). Encyclopedia of perennials. London, UK: Dorling Kindersley.

[ece32471-bib-0054] Richardson, D. M. , & Pyšek, P. (2012). Naturalization of introduced plants: Ecological drivers of biogeographical patterns. New Phytologist, 196, 383–396. 22943470 10.1111/j.1469-8137.2012.04292.x

[ece32471-bib-0055] Richardson, D. M. , Pyšek, P. , & Carlton, J. T. (2011). A compendium of essential concepts and terminology in invasion ecology In Richardson D. M. (Ed.), Fifty years of invasion ecology: The legacy of Charles Elton (pp. 409–420). Hoboken, NJ: Blackwell.

[ece32471-bib-0056] Royal Botanic Gardens Kew . (2015). Seed information database, version 7. Retrieved from http://data.kew.org/sid.

[ece32471-bib-0057] Schmidt, J. P. , & Drake, J. M. (2011). Time since introduction, seed mass, and genome size predict successful invaders among the cultivated vascular plants of Hawaii. PLoS ONE, 6, e17391. doi:doi.org/10.1371/journal.pone.0075078 21407804 10.1371/journal.pone.0017391 PMC3047568

[ece32471-bib-0058] Skou, A.‐M. T. , Pauleit, S. , & Kollmann, J. (2012). Tracing the introduction history of a potential invasive ornamental shrub: Variation in frost hardiness and climate change. Nordic Journal of Botany, 30, 739–746.

[ece32471-bib-0059] Stohlgren, T. J. , Pyšek, P. , Kartesz, J. , Nishino, M. , Pauchard, A. , Winter, M. , … Font, X. (2011). Widespread plant species: Natives versus aliens in our changing world. Biological Invasions, 13, 1931–1944.

[ece32471-bib-0060] Thiers, B. (2016). Index Herbariorum: a global directory of public herbaria and associated staff. Retrieved from http://sweetgum.nybg.org/science/ih.

[ece32471-bib-0061] Thompson, K. , & Davis, M. A. (2011). Why research on traits of invasive plants tells us very little. Trends in Ecology & Evolution, 26, 155–156. 21334760 10.1016/j.tree.2011.01.007

[ece32471-bib-0062] Thomson Reuters . (2013). Web of Science^™^ . Retrieved from http://wokinfo.com.

[ece32471-bib-0063] United States Department of Agriculture . (2015a). The PLANTS database. Retrieved from http://plants.usda.gov.

[ece32471-bib-0064] United States Department of Agriculture . (2015b). Germplasm Resources Information Network. Retrieved from http://www.ars-grin.gov.

[ece32471-bib-0065] United States Department of Agriculture . (2015c). United States Department of Agriculture plant hardiness zone map. Retrieved from http://planthardiness.ars.usda.gov.

[ece32471-bib-0066] van Kleunen, M. , Dawson, W. , & Dostal, P. (2011). Research on invasive‐plant traits tells us a lot. Trends in Ecology & Evolution, 26, 317. 21497938 10.1016/j.tree.2011.03.019

[ece32471-bib-0067] van Kleunen, M. , Dawson, W. , Essl, F. , Pergl, J. , Winter, M. , Weber, E. , … Pyšek, P. (2015). Global exchange and accumulation of non‐native plants. Nature, 525, 100–103. 26287466 10.1038/nature14910

[ece32471-bib-0068] van Kleunen, M. , Weber, E. , & Fischer, M. (2010). A meta‐analysis of trait differences between invasive and non‐invasive plant species. Ecology Letters, 13, 235–245. 20002494 10.1111/j.1461-0248.2009.01418.x

[ece32471-bib-0069] Vetaas, O. R. (2000). Comparing species temperature response curves: Population density versus second‐hand data. Journal of Vegetation Science, 11, 659–666.

[ece32471-bib-0070] Webb, C. O. , Ackerly, D. D. , & Kembel, S. W. (2008). Phylocom: Software for the analysis of phylogenetic community structure and trait evolution. Bioinformatics, 24, 2098–2100. 18678590 10.1093/bioinformatics/btn358

[ece32471-bib-0071] Webb, C. O. , & Donoghue, M. J. (2005). Phylomatic: Tree assembly for applied phylogenetics. Molecular Ecology Notes, 5, 181–183.

[ece32471-bib-0072] Wikström, N. , Savolainen, V. , & Chase, M. W. (2001). Evolution of the angiosperms: Calibrating the family tree. Proceedings of the Royal Society of London B: Biological Sciences, 268, 2211–2220. 10.1098/rspb.2001.1782 PMC1088868 11674868

[ece32471-bib-0073] Wu, S.‐H. , Rejmánek, M. , Grotkopp, E. , & DiTomaso, J. M. (2005). Herbarium records, actual distribution, and critical attributes of invasive plants: Genus *Crotalaria* in Taiwan. Taxon, 54, 133–138.

[ece32471-bib-0074] Zenni, R. D. , & Nuñez, M. A. (2013). The elephant in the room: The role of failed invasions in understanding invasion biology. Oikos, 122, 801–815.

