# Supplementary material for: Explaining naturalization and invasiveness: new insights from historical ornamental plant catalogs
Source: Ecol Evol. 2016 Sep 15;6(20):7188–98. doi: 10.1002/ece3.2471 (PMC5513274; doi:10.1002/ece3.2471)
Supplement: Supplementary file 1 [file ECE3-6-7188-s001.docx]

**Table S1.** Plant taxa listed in nursery catalogues published in Québec in the 19th century (outdoor plants only; species sold exclusively for human food production were discarded). Taxa still sold in 2015, naturalized and invasive (as defined in this study) in the province are indicated. Nomenclature follows the Canadian Biodiversity Information Facility (2015) or Tropicos (Missouri Botanical Garden, 2015) for taxa not listed in the former database.

| Species | Family | Sold in 2015 | Naturalized | Invasive |
| --- | --- | --- | --- | --- |
| *Abelmoschus moschatus* | Malvaceae | No | No | – |
| *Abronia latifolia* | Nyctaginaceae | No | No | – |
| *Abronia umbellata* | Nyctaginaceae | No | No | – |
| *Acanthus mollis* | Acanthaceae | Yes | No | – |
| *Acer campestre* | Sapindaceae | Yes | No | – |
| *Acer negundo* | Sapindaceae | Yes | Yes | Yes |
| *Acer platanoides* | Sapindaceae | Yes | Yes | Yes |
| *Acer pseudoplatanus* | Sapindaceae | No | No | – |
| *Achillea ptarmica* | Asteraceae | Yes | Yes | Yes |
| *Aconitum napellus* | Ranunculaceae | Yes | Yes | No |
| *Acorus calamus* | Acoraceae | Yes | Yes | Yes |
| *Actaea spicata* | Ranunculaceae | No | No | – |
| *Adonis aestivalis* | Ranunculaceae | No | No | – |
| *Adonis vernalis* | Ranunculaceae | No | No | – |
| *Aesculus flava* | Sapindaceae | No | No | – |
| *Aesculus glabra* var. *glabra* | Sapindaceae | Yes | No | – |
| *Aesculus hippocastanum* | Sapindaceae | Yes | Yes | No |
| *Aesculus parviflora* | Sapindaceae | No | No | – |
| *Aesculus pavia* | Sapindaceae | No | No | – |
| *Ageratum conyzoides* | Asteraceae | No | No | – |
| *Ageratum houstonianum* | Asteraceae | Yes | No | – |
| *Ageratum riparium* | Asteraceae | No | No | – |
| *Ailanthus altissima* | Simaroubaceae | Yes | Yes | No |
| *Aira tenorei* | Poaceae | No | No | – |
| *Ajuga genevensis* | Lamiaceae | No | Yes | No |
| *Ajuga pyramidalis* | Lamiaceae | Yes | No | – |
| *Alcea rosea* | Malvaceae | Yes | Yes | No |
| *Allium moly* | Amaryllidaceae | Yes | No | – |
| *Alonsoa meridionalis* | Scrophulariaceae | No | No | – |
| *Alyssum wierzbickii* | Brassicaceae | No | No | – |
| *Amaranthus atropurpureus* | Amaranthaceae | No | No | – |
| *Amaranthus caudatus* | Amaranthaceae | Yes | Yes | No |
| *Amaranthus cruentus* | Amaranthaceae | Yes | Yes | No |
| *Amaranthus tricolor* | Amaranthaceae | No | No | – |
| *Amberboa moschata* | Asteraceae | No | No | – |
| *Amorpha fruticosa* | Fabaceae | Yes | Yes | No |
| *Amsonia tabernaemontana* | Apocynaceae | Yes | No | – |
| *Anagallis monelli* | Primulaceae | No | No | – |
| *Anchusa azurea* | Boraginaceae | Yes | No | – |
| *Anemone alpina* | Ranunculaceae | No | No | – |
| *Anemone hortensis* | Ranunculaceae | No | No | – |
| *Anemone nemorosa* | Ranunculaceae | No | Yes | No |
| *Anemone pulsatilla* | Ranunculaceae | Yes | No | – |
| *Anemone* ×*fulgens* | Ranunculaceae | No | No | – |
| *Angelica archangelica* | Apiaceae | Yes | No | – |
| *Antirrhinum majus* | Scrophulariaceae | Yes | Yes | No |
| *Aptenia cordifolia* | Aizoaceae | No | No | – |
| *Aquilegia chrysantha* | Ranunculaceae | Yes | No | – |
| *Aquilegia glandulosa* | Ranunculaceae | No | No | – |
| *Aquilegia viridiflora* | Ranunculaceae | No | No | – |
| *Aquilegia vulgaris* | Ranunculaceae | Yes | Yes | Yes |
| *Aralia spinosa* | Araliaceae | Yes | No | – |
| *Arctotheca calendula* | Asteraceae | No | No | – |
| *Argemone mexicana* | Papaveraceae | No | Yes | No |
| *Aristolochia clematitis* | Aristolochiaceae | No | Yes | No |
| *Aristolochia macrophylla* | Aristolochiaceae | No | Yes | No |
| *Artemisia scoparia* | Asteraceae | No | No | – |
| *Arum maculatum* | Araceae | No | No | – |
| *Aruncus dioicus* var*. vulgaris* | Rosaceae | Yes | Yes | No |
| *Arundo donax* | Poaceae | Yes | No | – |
| *Asparagus asparagoides* | Asparagaceae | No | No | – |
| *Asperula orientalis* | Rubiaceae | No | No | – |
| *Asphodelus albus* | Xanthorrhoeaceae | No | No | – |
| *Astragalus alopecuroides* | Fabaceae | No | No | – |
| *Aubrieta deltoidea* | Brassicaceae | No | No | – |
| *Aureolaria virginica* | Orobanchaceae | No | No | – |
| *Aurinia saxatilis* | Brassicaceae | Yes | Yes | No |
| *Avena sterilis* | Poaceae | No | No | – |
| *Baptisia alba* | Fabaceae | Yes | No | – |
| *Baptisia australis* | Fabaceae | Yes | No | – |
| *Baptisia tinctoria* | Fabaceae | Yes | No | – |
| *Barbarea vulgaris* | Brassicaceae | No | Yes | Yes |
| *Begonia froebelii* | Begoniaceae | No | No | – |
| *Begonia pearcei* | Begoniaceae | No | No | – |
| *Begonia veitchii* | Begoniaceae | No | No | – |
| *Bellis perennis* | Asteraceae | Yes | Yes | No |
| *Berberis lutea* | Berberidaceae | No | No | – |
| *Berberis vulgaris* | Berberidaceae | No | Yes | Yes |
| *Bergenia crassifolia* | Saxifragaceae | Yes | No | – |
| *Beta vulgaris* subsp. *cicla* | Amaranthaceae | Yes | No | – |
| *Betula pendula* | Betulaceae | Yes | Yes | No |
| *Bignonia capreolata* | Bignoniaceae | No | No | – |
| *Blennosperma nanum* | Asteraceae | No | No | – |
| *Briza maxima* | Poaceae | No | Yes | No |
| *Briza minor* | Poaceae | No | No | – |
| *Bromus briziformis* | Poaceae | No | Yes | No |
| *Browallia americana* | Solanaceae | No | No | – |
| *Buxus sempervirens* | Buxaceae | No | No | – |
| *Calceolaria integrifolia* | Calceolariaceae | No | No | – |
| *Calceolaria tripartita* | Calceolariaceae | No | No | – |
| *Calceolaria* ×*herbeohybrida* | Calceolariaceae | No | No | – |
| *Calendula officinalis* | Asteraceae | Yes | Yes | No |
| *Callirhoe involucrata* | Malvaceae | Yes | No | – |
| *Callirhoe pedata* | Malvaceae | No | No | – |
| *Callistephus chinensis* | Asteraceae | Yes | Yes | No |
| *Calluna vulgaris* | Ericaceae | Yes | Yes | No |
| *Calycanthus floridus* | Calycanthaceae | Yes | No | – |
| *Campanula carpatica* | Campanulaceae | Yes | No | – |
| *Campanula medium* | Campanulaceae | Yes | Yes | No |
| *Campanula persicifolia* | Campanulaceae | Yes | Yes | No |
| *Campanula punctata* | Campanulaceae | Yes | No | – |
| *Campanula pyramidalis* | Campanulaceae | Yes | No | – |
| *Campanula ramosissima* | Campanulaceae | No | No | – |
| *Campanula sibirica* | Campanulaceae | No | No | – |
| *Campanula trachelium* | Campanulaceae | No | Yes | No |
| *Campsis grandiflora* | Bignoniaceae | No | No | – |
| *Campsis radicans* | Bignoniaceae | Yes | No | – |
| *Canna glauca* | Cannaceae | No | No | – |
| *Canna indica* | Cannaceae | No | No | – |
| *Canna tuerckheimii* | Cannaceae | No | No | – |
| *Caragana arborescens* | Fabaceae | Yes | Yes | Yes |
| *Cardamine pratensis* | Brassicaceae | No | Yes | No |
| *Cardiospermum halicacabum* | Sapindaceae | No | No | – |
| *Carya tomentosa* | Juglandaceae | No | No | – |
| *Castanea dentata* | Fagaceae | Yes | Yes | No |
| *Catalpa bignonioides* | Bignoniaceae | Yes | Yes | No |
| *Catalpa speciosa* | Bignoniaceae | Yes | Yes | No |
| *Catananche caerulea* | Asteraceae | Yes | No | – |
| *Catharanthus roseus* | Apocynaceae | No | No | – |
| *Cedrus libani* | Pinaceae | No | No | – |
| *Celosia cristata* | Amaranthaceae | Yes | No | – |
| *Cenchrus clandestinus* | Poaceae | No | No | – |
| *Centaurea babylonica* | Asteraceae | No | No | – |
| *Centaurea cineraria* | Asteraceae | No | No | – |
| *Centaurea clementei* | Asteraceae | No | No | – |
| *Centaurea depressa* | Asteraceae | No | No | – |
| *Centaurea rutifolia* | Asteraceae | No | No | – |
| *Centranthus macrosiphon* | Caprifoliaceae | No | No | – |
| *Centranthus ruber* | Caprifoliaceae | Yes | No | – |
| *Cercis canadensis* | Fabaceae | Yes | No | – |
| *Chaenomeles japonica* | Rosaceae | Yes | Yes | No |
| *Chamaecytisus hirsutus* | Fabaceae | No | No | – |
| *Chamaemelum nobile* | Asteraceae | Yes | No | – |
| *Chelone obliqua* | Plantaginaceae | Yes | No | – |
| *Cistanthe grandiflora* | Montiaceae | No | No | – |
| *Cistanthe umbellata* | Montiaceae | No | No | – |
| *Clarkia amoena* subsp. *amoena* | Onagraceae | No | Yes | No |
| *Clarkia amoena* subsp. *whitneyi* | Onagraceae | No | No | – |
| *Clarkia pulchella* | Onagraceae | No | No | – |
| *Clarkia rubicunda* | Onagraceae | No | No | – |
| *Clarkia unguiculata* | Onagraceae | No | No | – |
| *Clematis crispa* | Ranunculaceae | No | No | – |
| *Clematis flammula* | Ranunculaceae | No | No | – |
| *Clematis integrifolia* | Ranunculaceae | Yes | No | – |
| *Clematis patens* | Ranunculaceae | No | No | – |
| *Clematis recta* | Ranunculaceae | No | No | – |
| *Clematis terniflora* | Ranunculaceae | No | No | – |
| *Clematis viorna* | Ranunculaceae | No | No | – |
| *Clematis vitalba* | Ranunculaceae | No | No | – |
| *Clematis viticella* | Ranunculaceae | Yes | Yes | No |
| *Cobaea scandens* | Polemoniaceae | Yes | No | – |
| *Coix lacryma-jobi* | Poaceae | No | No | – |
| *Colchicum autumnale* | Colchicaceae | No | Yes | No |
| *Colchicum variegatum* | Colchicaceae | No | No | – |
| *Collinsia heterophylla* | Plantaginaceae | No | No | – |
| *Collomia biflora* | Polemoniaceae | No | No | – |
| *Colutea arborescens* | Fabaceae | No | No | – |
| *Commelina tuberosa* | Commelinaceae | Yes | No | – |
| *Conoclinium coelestinum* | Asteraceae | No | No | – |
| *Consolida ajacis* | Ranunculaceae | No | Yes | No |
| *Consolida regalis* | Ranunculaceae | No | No | – |
| *Convallaria majalis* | Asparagaceae | Yes | Yes | No |
| *Convolvulus tricolor* | Convolvulaceae | No | No | – |
| *Coreopsis tinctoria* | Asteraceae | Yes | Yes | No |
| *Coreopsis tripteris* | Asteraceae | Yes | Yes | No |
| *Cornus florida* | Cornaceae | No | No | – |
| *Cornus sanguinea* | Cornaceae | Yes | No | – |
| *Corylus avellana* | Betulaceae | Yes | No | – |
| *Cotinus coggygria* | Anacardiaceae | Yes | No | – |
| *Crocus vernus* | Iridaceae | Yes | No | – |
| *Cuphea hookeriana* | Lythraceae | No | No | – |
| *Cuphea ignea* | Lythraceae | No | No | – |
| *Cuphea lanceolata* | Lythraceae | No | No | – |
| *Cuphea micropetala* | Lythraceae | No | No | – |
| *Cuphea strigulosa* | Lythraceae | No | No | – |
| *Cyclamen persicum* | Primulaceae | No | No | – |
| *Cyclamen purpurascens* | Primulaceae | No | No | – |
| *Cyclanthera brachystachya* | Cucurbitaceae | No | No | – |
| *Cymbalaria muralis* | Scrophulariaceae | Yes | Yes | No |
| *Cytisus scoparius* | Fabaceae | No | No | – |
| *Daphne mezereum* | Thymelaeaceae | Yes | Yes | No |
| *Datura metel* | Solanaceae | Yes | Yes | No |
| *Delphinium carolinianum* subsp. *carolinianum* | Ranunculaceae | No | No | – |
| *Delphinium elatum* | Ranunculaceae | Yes | Yes | No |
| *Delphinium exaltatum* | Ranunculaceae | No | No | – |
| *Delphinium formosum* | Ranunculaceae | No | No | – |
| *Delphinium grandiflorum* | Ranunculaceae | Yes | No | – |
| *Delphinium nudicaule* | Ranunculaceae | Yes | No | – |
| *Delphinium verdunense* | Ranunculaceae | No | No | – |
| *Dendranthema indicum* | Asteraceae | No | No | – |
| *Desmazeria sicula* | Poaceae | No | No | – |
| *Deutzia scabra* | Hydrangeaceae | No | No | – |
| *Dianthus barbatus* | Caryophyllaceae | Yes | Yes | No |
| *Dianthus caryophyllus* | Caryophyllaceae | Yes | No | – |
| *Dianthus chinensis* | Caryophyllaceae | Yes | No | – |
| *Dianthus plumarius* | Caryophyllaceae | Yes | Yes | No |
| *Dicentra formosa* | Papaveraceae | Yes | No | – |
| *Dicentra spectabilis* | Papaveraceae | Yes | No | – |
| *Dictamnus albus* | Rutaceae | Yes | No | – |
| *Digitalis grandiflora* | Scrophulariaceae | Yes | No | – |
| *Digitalis lutea* | Scrophulariaceae | No | Yes | No |
| *Digitalis purpurea* | Scrophulariaceae | Yes | Yes | No |
| *Dorotheanthus bellidiformis* | Aizoaceae | No | No | – |
| *Downingia pulchella* | Campanulaceae | No | No | – |
| *Dracocephalum imberbe* | Lamiaceae | No | No | – |
| *Dracocephalum moldavica* | Lamiaceae | No | No | – |
| *Dracunculus vulgaris* | Araceae | No | No | – |
| *Echinacea purpurea* | Asteraceae | Yes | Yes | No |
| *Emilia coccinea* | Asteraceae | No | No | – |
| *Eragrostis japonica* | Poaceae | No | No | – |
| *Erica herbacea* | Ericaceae | No | No | – |
| *Erica vagans* | Ericaceae | No | No | – |
| *Erigeron grandiflorus* | Asteraceae | Yes | No | – |
| *Erodium moschatum* | Geraniaceae | No | Yes | No |
| *Eryngium maritimum* | Apiaceae | No | No | – |
| *Eryngium planum* | Apiaceae | Yes | Yes | No |
| *Erysimum capitatum* | Brassicaceae | No | No | – |
| *Erysimum cheiri* | Brassicaceae | No | Yes | No |
| *Erysimum perofskianum* | Brassicaceae | No | No | – |
| *Erythrina crista-galli* | Fabaceae | No | No | – |
| *Eschscholzia californica* | Papaveraceae | Yes | No | – |
| *Euphorbia lathyris* | Euphorbiaceae | No | Yes | No |
| *Euphorbia marginata* | Euphorbiaceae | No | Yes | No |
| *Eustoma exaltatum* subsp. *russellianum* | Gentianaceae | No | No | – |
| *Fagus sylvatica* | Fagaceae | Yes | No | – |
| *Felicia heterophylla* | Asteraceae | No | No | – |
| *Festuca glauca* | Poaceae | Yes | No | – |
| *Filipendula ulmaria* | Rosaceae | Yes | Yes | Yes |
| *Filipendula vulgaris* | Rosaceae | Yes | No | – |
| *Fraxinus excelsior* | Oleaceae | Yes | No | – |
| *Fraxinus ornus* | Oleaceae | No | No | – |
| *Fritillaria imperialis* | Liliaceae | No | No | – |
| *Fritillaria meleagris* | Liliaceae | No | No | – |
| *Fritillaria persica* | Liliaceae | No | No | – |
| *Gagea lutea* | Liliaceae | No | No | – |
| *Gaillardia pulchella* | Asteraceae | Yes | Yes | No |
| *Gaillardia* ×*grandiflora* | Asteraceae | Yes | No | – |
| *Galanthus nivalis* | Amaryllidaceae | Yes | No | – |
| *Galega officinalis* | Fabaceae | No | Yes | No |
| *Galium odoratum* | Rubiaceae | Yes | Yes | No |
| *Gaylussacia frondosa* | Ericaceae | No | No | – |
| *Gentiana acaulis* | Gentianaceae | No | No | – |
| *Gentiana saponaria* | Gentianaceae | No | No | – |
| *Geranium macrorrhizum* | Geraniaceae | Yes | No | – |
| *Geranium pratense* | Geraniaceae | Yes | Yes | Yes |
| *Geranium sanguineum* | Geraniaceae | Yes | Yes | No |
| *Geum coccineum* | Rosaceae | No | No | – |
| *Gilia tricolor* | Polemoniaceae | No | No | – |
| *Ginkgo biloba* | Ginkgoaceae | Yes | No | – |
| *Gladiolus communis* | Iridaceae | No | No | – |
| *Glandularia platensis* | Verbenaceae | No | No | – |
| *Glandularia* ×*hybrida* | Verbenaceae | No | No | – |
| *Glaucium flavum* | Papaveraceae | No | No | – |
| *Glebionis coronarium* | Asteraceae | No | No | – |
| *Glechoma hederacea* | Lamiaceae | Yes | Yes | Yes |
| *Gleditsia triacanthos* | Fabaceae | Yes | Yes | No |
| *Gunnera tinctoria* | Gunneraceae | No | No | – |
| *Gymnocladus dioicus* | Fabaceae | Yes | Yes | No |
| *Gypsophila muralis* | Caryophyllaceae | Yes | Yes | No |
| *Gypsophila paniculata* | Caryophyllaceae | Yes | Yes | No |
| *Halesia carolina* | Styracaceae | Yes | No | – |
| *Hedera helix* | Araliaceae | Yes | No | – |
| *Hedysarum coronarium* | Fabaceae | No | No | – |
| *Helianthus annuus* | Asteraceae | Yes | Yes | Yes |
| *Helianthus giganteus* | Asteraceae | No | Yes | No |
| *Helianthus salicifolius* | Asteraceae | No | No | – |
| *Helianthus tuberosus* | Asteraceae | Yes | Yes | Yes |
| *Helianthus* ×*multiflorus* | Asteraceae | No | No | – |
| *Helichrysum foetidum* | Asteraceae | No | No | – |
| *Heliotropium nicotianifolium* | Boraginaceae | No | No | – |
| *Helleborus niger* | Ranunculaceae | Yes | No | – |
| *Hemerocallis lilioasphodelus* | Xanthorrhoeaceae | Yes | Yes | No |
| *Hemerocallis minor* | Xanthorrhoeaceae | No | No | – |
| *Hesperis matronalis* | Brassicaceae | Yes | Yes | Yes |
| *Hibiscus grandiflorus* | Malvaceae | No | No | – |
| *Hibiscus laevis* | Malvaceae | No | No | – |
| *Hibiscus moscheutos* subsp*. moscheutos* | Malvaceae | Yes | No | – |
| *Hibiscus syriacus* | Malvaceae | Yes | No | – |
| *Hosta plantaginea* | Asparagaceae | Yes | Yes | No |
| *Hosta ventricosa* | Asparagaceae | Yes | No | – |
| *Hyacinthus orientalis* | Asparagaceae | No | No | – |
| *Hydrangea arborescens* | Hydrangeaceae | Yes | Yes | No |
| *Hydrangea macrophylla* | Hydrangeaceae | Yes | No | – |
| *Hydrangea paniculata* | Hydrangeaceae | Yes | Yes | No |
| *Hydrangea quercifolia* | Hydrangeaceae | Yes | No | – |
| *Hydrangea radiata* | Hydrangeaceae | No | No | – |
| *Hylotelephium populifolium* | Crassulaceae | No | No | – |
| *Hylotelephium telephioides* | Crassulaceae | No | No | – |
| *Hylotelephium telephium* | Crassulaceae | No | Yes | Yes |
| *Hypericum prolificum* | Hypericaceae | No | No | – |
| *Hyssopus officinalis* | Lamiaceae | Yes | Yes | No |
| *Iberis amara* | Brassicaceae | No | Yes | No |
| *Iberis carnosa* | Brassicaceae | No | No | – |
| *Iberis odorata* | Brassicaceae | No | No | – |
| *Iberis semperflorens* | Brassicaceae | No | No | – |
| *Iberis sempervirens* | Brassicaceae | Yes | No | – |
| *Iberis umbellata* | Brassicaceae | No | Yes | No |
| *Ilex aquifolium* | Aquifoliaceae | No | No | – |
| *Impatiens balsamina* | Balsaminaceae | Yes | Yes | No |
| *Inula helenium* | Asteraceae | No | Yes | Yes |
| *Ipomoea pandurata* | Convolvulaceae | No | No | – |
| *Ipomoea quamoclit* | Convolvulaceae | No | No | – |
| *Ipomopsis rubra* | Polemoniaceae | Yes | No | – |
| *Iris aphylla* | Iridaceae | No | No | – |
| *Iris domestica* | Iridaceae | No | No | – |
| *Iris foetidissima* | Iridaceae | No | No | – |
| *Iris fulva* | Iridaceae | No | No | – |
| *Iris germanica* | Iridaceae | Yes | Yes | No |
| *Iris graminea* | Iridaceae | No | No | – |
| *Iris latifolia* | Iridaceae | No | No | – |
| *Iris missouriensis* | Iridaceae | No | No | – |
| *Iris pallida* | Iridaceae | Yes | No | – |
| *Iris persica* | Iridaceae | No | No | – |
| *Iris pseudacorus* | Iridaceae | Yes | Yes | No |
| *Iris pumila* | Iridaceae | Yes | Yes | No |
| *Iris sibirica* | Iridaceae | Yes | Yes | No |
| *Iris spuria* | Iridaceae | No | No | – |
| *Iris spuria* subsp. *ochroleuca* | Iridaceae | No | No | – |
| *Iris susiana* | Iridaceae | No | No | – |
| *Iris verna* var*. verna* | Iridaceae | No | No | – |
| *Iris xiphium* | Iridaceae | No | No | – |
| *Ismelia carinata* | Asteraceae | No | No | – |
| *Jacobaea maritima* | Asteraceae | No | No | – |
| *Jasminum officinale* | Oleaceae | No | No | – |
| *Juglans nigra* | Juglandaceae | Yes | Yes | No |
| *Juniperus sabina* | Cupressaceae | Yes | Yes | No |
| *Kalimeris incisa* | Asteraceae | No | No | – |
| *Kerria japonica* | Rosaceae | Yes | No | – |
| *Kochia scoparia* | Amaranthaceae | Yes | Yes | Yes |
| *Lablab purpureus* | Fabaceae | No | No | – |
| *Laburnum anagyroides* | Fabaceae | No | No | – |
| *Lagenaria siceraria* | Cucurbitaceae | No | No | – |
| *Lagerstroemia indica* | Lythraceae | No | No | – |
| *Lagurus ovatus* | Poaceae | Yes | No | – |
| *Lallemantia canescens* | Lamiaceae | No | No | – |
| *Lamium album* | Lamiaceae | Yes | Yes | No |
| *Larix decidua* | Pinaceae | Yes | Yes | No |
| *Lasthenia coronaria* | Asteraceae | No | No | – |
| *Lathyrus latifolius* | Fabaceae | Yes | Yes | Yes |
| *Lathyrus niger* | Fabaceae | No | No | – |
| *Lathyrus odoratus* | Fabaceae | Yes | No | – |
| *Lathyrus vernus* | Fabaceae | Yes | No | – |
| *Lavatera trimestris* | Malvaceae | Yes | Yes | No |
| *Legousia speculum-veneris* | Campanulaceae | No | No | – |
| *Lessertia frutescens* | Fabaceae | No | No | – |
| *Leucojum vernum* | Amaryllidaceae | No | No | – |
| *Liatris scariosa* | Asteraceae | Yes | No | – |
| *Liatris spicata* | Asteraceae | Yes | Yes | No |
| *Liatris squarrosa* | Asteraceae | No | No | – |
| *Lilium bulbiferum* | Liliaceae | No | Yes | No |
| *Lilium candidum* | Liliaceae | No | No | – |
| *Lilium catesbaei* | Liliaceae | No | No | – |
| *Lilium chalcedonicum* | Liliaceae | No | No | – |
| *Lilium concolor* | Liliaceae | No | No | – |
| *Lilium japonicum* | Liliaceae | No | No | – |
| *Lilium martagon* | Liliaceae | Yes | Yes | No |
| *Lilium pomponium* | Liliaceae | No | No | – |
| *Lilium superbum* | Liliaceae | No | No | – |
| *Limnanthes douglasii* | Limnanthaceae | No | No | – |
| *Limonium sinuatum* | Plumbaginaceae | Yes | No | – |
| *Linaria bipartita* | Scrophulariaceae | No | No | – |
| *Lindelofia longiflora* | Boraginaceae | No | No | – |
| *Linum grandiflorum* | Linaceae | No | No | – |
| *Linum perenne* | Linaceae | Yes | Yes | No |
| *Liquidambar styraciflua* | Altingiaceae | Yes | No | – |
| *Liriodendron tulipifera* | Magnoliaceae | Yes | No | – |
| *Lobelia erinus* | Campanulaceae | Yes | No | – |
| *Lobelia siphilitica* | Campanulaceae | Yes | No | – |
| *Lobularia maritima* | Brassicaceae | No | Yes | No |
| *Lonicera caprifolium* | Caprifoliaceae | No | No | – |
| *Lonicera flava* | Caprifoliaceae | No | No | – |
| *Lonicera japonica* | Caprifoliaceae | Yes | No | – |
| *Lonicera periclymenum* | Caprifoliaceae | Yes | No | – |
| *Lonicera sempervirens* | Caprifoliaceae | Yes | Yes | No |
| *Lonicera tatarica* | Caprifoliaceae | Yes | Yes | Yes |
| *Lonicera xylosteum* | Caprifoliaceae | No | Yes | No |
| *Lophospermum scandens* | Plantaginaceae | No | No | – |
| *Lotus jacobaeus* | Fabaceae | No | No | – |
| *Lunaria annua* | Brassicaceae | Yes | Yes | No |
| *Lupinus hartwegii* | Fabaceae | No | No | – |
| *Lupinus perennis* | Fabaceae | Yes | Yes | No |
| *Lupinus polyphyllus* | Fabaceae | Yes | Yes | No |
| *Lupinus pubescens* | Fabaceae | No | No | – |
| *Lupinus subcarnosus* | Fabaceae | No | No | – |
| *Lupinus tomentosus* | Fabaceae | No | No | – |
| *Lycium barbarum* | Solanaceae | Yes | Yes | No |
| *Lyonia ligustrina* | Ericaceae | No | No | – |
| *Lyonia mariana* | Ericaceae | No | No | – |
| *Lythrum salicaria* | Lythraceae | Yes | Yes | Yes |
| *Macleaya cordata* | Papaveraceae | Yes | Yes | No |
| *Maclura pomifera* | Moraceae | No | No | – |
| *Magnolia acuminata* | Magnoliaceae | Yes | No | – |
| *Magnolia denudata* | Magnoliaceae | No | No | – |
| *Magnolia liliiflora* | Magnoliaceae | No | No | – |
| *Magnolia macrophylla* | Magnoliaceae | No | No | – |
| *Magnolia tripetala* | Magnoliaceae | No | No | – |
| *Magnolia virginiana* | Magnoliaceae | No | No | – |
| *Malcolmia maritima* | Brassicaceae | No | Yes | No |
| *Malope trifida* | Malvaceae | No | No | – |
| *Matthiola incana* | Brassicaceae | Yes | No | – |
| *Matthiola tricuspidata* | Brassicaceae | No | No | – |
| *Maurandya barclaiana* | Plantaginaceae | No | No | – |
| *Melinis repens* | Poaceae | No | No | – |
| *Mentzelia lindleyi* | Loasaceae | No | No | – |
| *Mesembryanthemum crystallinum* | Aizoaceae | No | No | – |
| *Mespilus germanica* | Rosaceae | No | No | – |
| *Mimosa pudica* | Fabaceae | Yes | No | – |
| *Mimulus cardinalis* | Scrophulariaceae | No | No | – |
| *Mimulus cupreus* | Scrophulariaceae | No | No | – |
| *Mimulus guttatus* | Scrophulariaceae | No | No | – |
| *Mimulus luteus* | Scrophulariaceae | No | No | – |
| *Mimulus moschatus* | Scrophulariaceae | No | Yes | No |
| *Mirabilis jalapa* | Nyctaginaceae | Yes | No | – |
| *Momordica balsamina* | Cucurbitaceae | No | No | – |
| *Monarda didyma* | Lamiaceae | Yes | Yes | No |
| *Montiopsis umbellata* | Montiaceae | No | No | – |
| *Morus alba* | Moraceae | Yes | Yes | No |
| *Muscari neglectum* | Asparagaceae | No | No | – |
| *Myosotis arvensis* | Boraginaceae | No | Yes | Yes |
| *Myosotis azorica* | Boraginaceae | No | No | – |
| *Myosotis scorpioides* | Boraginaceae | Yes | Yes | Yes |
| *Narcissus jonquilla* | Amaryllidaceae | No | No | – |
| *Narcissus poeticus* | Amaryllidaceae | No | Yes | No |
| *Narcissus tazetta* | Amaryllidaceae | No | No | – |
| *Nasa triphylla* | Loasaceae | No | No | – |
| *Nemesia floribunda* | Scrophulariaceae | No | No | – |
| *Nemophila maculata* | Boraginaceae | No | No | – |
| *Nemophila menziesii* var*. menziesii* | Boraginaceae | No | No | – |
| *Nierembergia gracilis* | Solanaceae | No | No | – |
| *Nierembergia scoparia* | Solanaceae | No | No | – |
| *Nigella hispanica* | Ranunculaceae | No | No | – |
| *Nolana humifusa* | Solanaceae | No | No | – |
| *Nyssa sylvatica* | Cornaceae | Yes | No | – |
| *Oenothera acaulis* | Onagraceae | No | No | – |
| *Oenothera drummondii* | Onagraceae | No | No | – |
| *Oenothera grandiflora* | Onagraceae | No | Yes | No |
| *Oenothera lindheimeri* | Onagraceae | Yes | No | – |
| *Oenothera macrocarpa* subsp*. macrocarpa* | Onagraceae | Yes | No | – |
| *Omphalodes linifolia* | Boraginaceae | No | No | – |
| *Omphalodes verna* | Boraginaceae | No | Yes | No |
| *Ononis rotundifolia* | Fabaceae | No | No | – |
| *Onopordum alexandrinum* | Asteraceae | No | No | – |
| *Opuntia fragilis* | Cactaceae | Yes | No | – |
| *Opuntia humifusa* var*. humifusa* | Cactaceae | Yes | No | – |
| *Ornithogalum narbonense* | Liliaceae | No | No | – |
| *Ornithogalum umbellatum* | Liliaceae | Yes | Yes | No |
| *Oxalis corniculata* | Oxalidaceae | No | Yes | No |
| *Oxalis rosea* | Oxalidaceae | No | No | – |
| *Oxalis valdiviensis* | Oxalidaceae | No | No | – |
| *Oxypetalum coeruleum* | Apocynaceae | No | No | – |
| *Paeonia anomala* | Paeoniaceae | No | No | – |
| *Paeonia lactiflora* | Paeoniaceae | Yes | No | – |
| *Paeonia officinalis* | Paeoniaceae | Yes | Yes | No |
| *Paeonia officinalis* subsp*. microcarpa* | Paeoniaceae | Yes | No | – |
| *Paeonia suffruticosa* | Paeoniaceae | Yes | No | – |
| *Paeonia tenuifolia* | Paeoniaceae | Yes | No | – |
| *Papaver bracteatum* | Papaveraceae | No | No | – |
| *Papaver rhoeas* | Papaveraceae | No | Yes | No |
| *Papaver somniferum* | Papaveraceae | No | Yes | No |
| *Paradisea liliastrum* | Asparagaceae | No | No | – |
| *Parthenocissus quinquefolia* | Vitaceae | Yes | Yes | Yes |
| *Penstemon barbatus* var*. barbatus* | Scrophulariaceae | Yes | No | – |
| *Penstemon hartwegii* | Scrophulariaceae | Yes | No | – |
| *Pentaglottis sempervirens* | Boraginaceae | No | No | – |
| *Pericallis hybrida* | Asteraceae | No | No | – |
| *Perilla frutescens* var*. crispa* | Lamiaceae | No | No | – |
| *Persicaria orientalis* | Polygonaceae | No | Yes | No |
| *Petunia hybrida* | Solanaceae | Yes | Yes | No |
| *Petunia integrifolia* | Solanaceae | No | No | – |
| *Phacelia congesta* | Boraginaceae | No | No | – |
| *Phacelia menziesii* | Boraginaceae | No | No | – |
| *Phacelia minor* | Boraginaceae | No | No | – |
| *Phacelia viscida* | Boraginaceae | No | No | – |
| *Phalaris arundinacea* | Poaceae | Yes | Yes | Yes |
| *Phaseolus coccineus* | Fabaceae | No | No | – |
| *Phedimus aizoon* | Crassulaceae | No | Yes | No |
| *Philadelphus coronarius* | Hydrangeaceae | Yes | Yes | No |
| *Philadelphus inodorus* | Hydrangeaceae | No | No | – |
| *Phlomis tuberosa* | Lamiaceae | No | No | – |
| *Phlox acuminata* | Polemoniaceae | No | No | – |
| *Phlox drummondii* | Polemoniaceae | Yes | Yes | No |
| *Phlox latifolia* | Polemoniaceae | No | No | – |
| *Phlox maculata* | Polemoniaceae | Yes | Yes | No |
| *Phlox paniculata* | Polemoniaceae | Yes | Yes | No |
| *Phlox subulata* subsp*. australis* | Polemoniaceae | Yes | No | – |
| *Picea abies* | Pinaceae | Yes | Yes | No |
| *Pinus echinata* | Pinaceae | No | No | – |
| *Pinus sylvestris* | Pinaceae | Yes | Yes | Yes |
| *Piptatherum miliaceum* | Poaceae | No | No | – |
| *Platanthera bifolia* | Orchidaceae | No | No | – |
| *Platycladus orientalis* | Cupressaceae | No | No | – |
| *Platycodon grandiflorus* | Campanulaceae | Yes | No | – |
| *Podachaenium eminens* | Asteraceae | No | No | – |
| *Polemonium caeruleum* | Polemoniaceae | Yes | Yes | No |
| *Polygonatum odoratum* var*. odoratum* | Asparagaceae | No | No | – |
| *Populus alba* | Salicaceae | No | Yes | Yes |
| *Populus deltoides* subsp*. monilifera* | Salicaceae | No | No | – |
| *Populus nigra* | Salicaceae | Yes | Yes | No |
| *Portulaca grandiflora* | Portulacaceae | Yes | Yes | No |
| *Potentilla atrosanguinea* | Rosaceae | Yes | No | – |
| *Potentilla canadensis* | Rosaceae | No | No | – |
| *Potentilla grandiflora* | Rosaceae | No | No | – |
| *Potentilla nepalensis* | Rosaceae | Yes | No | – |
| *Primula auricula* | Primulaceae | Yes | No | – |
| *Primula cortusoides* | Primulaceae | Yes | No | – |
| *Primula elatior* | Primulaceae | Yes | No | – |
| *Primula japonica* | Primulaceae | Yes | No | – |
| *Primula macrophylla* var*. macrophylla* | Primulaceae | No | No | – |
| *Primula meadia* | Primulaceae | No | No | – |
| *Primula praenitens* | Primulaceae | No | No | – |
| *Primula veris* | Primulaceae | No | Yes | No |
| *Primula vulgaris* | Primulaceae | Yes | No | – |
| *Primula* ×*polyantha* | Primulaceae | Yes | No | – |
| *Proboscidea louisianica* subsp*. fragrans* | Martyniaceae | No | No | – |
| *Prunus domestica* | Rosaceae | Yes | Yes | No |
| *Prunus fruticosa* | Rosaceae | No | No | – |
| *Prunus padus* | Rosaceae | Yes | No | – |
| *Prunus tomentosa* | Rosaceae | Yes | No | – |
| *Pulmonaria officinalis* | Boraginaceae | No | Yes | No |
| *Pycnanthemum montanum* | Lamiaceae | No | No | – |
| *Pyracantha coccinea* | Rosaceae | Yes | No | – |
| *Pyrus nivalis* | Rosaceae | No | No | – |
| *Pyrus salicifolia* | Rosaceae | Yes | No | – |
| *Pyrus spinosa* | Rosaceae | No | No | – |
| *Ranunculus aconitifolius* | Ranunculaceae | No | No | – |
| *Ranunculus acris* | Ranunculaceae | No | Yes | Yes |
| *Ranunculus bulbosus* | Ranunculaceae | No | Yes | No |
| *Ranunculus repens* | Ranunculaceae | No | Yes | Yes |
| *Reseda odorata* | Resedaceae | No | Yes | No |
| *Rheum australe* | Polygonaceae | No | No | – |
| *Rheum compactum* | Polygonaceae | No | No | – |
| *Rheum palmatum* | Polygonaceae | Yes | No | – |
| *Rheum rhabarbarum* | Polygonaceae | Yes | Yes | No |
| *Rheum rhaponticum* | Polygonaceae | No | No | – |
| *Rheum tataricum* | Polygonaceae | No | No | – |
| *Rhododendron catawbiense* | Ericaceae | Yes | No | – |
| *Rhododendron maximum* | Ericaceae | No | No | – |
| *Rhododendron minus* var. *minus* | Ericaceae | No | No | – |
| *Rhododendron periclymenoides* | Ericaceae | No | No | – |
| *Rhododendron ponticum* | Ericaceae | No | No | – |
| *Rhus glabra* | Anacardiaceae | Yes | No | – |
| *Ricinus communis* | Euphorbiaceae | Yes | No | – |
| *Robinia hispida* | Fabaceae | Yes | Yes | No |
| *Robinia pseudoacacia* | Fabaceae | Yes | Yes | Yes |
| *Robinia viscosa* | Fabaceae | No | Yes | No |
| *Rosa banksiae* | Rosaceae | No | No | – |
| *Rosa brunonii* | Rosaceae | No | No | – |
| *Rosa chinensis* | Rosaceae | No | No | – |
| *Rosa chinensis* var*. semperflorens* | Rosaceae | No | No | – |
| *Rosa foetida* | Rosaceae | Yes | No | – |
| *Rosa gallica* | Rosaceae | No | Yes | No |
| *Rosa indica* | Rosaceae | No | No | – |
| *Rosa laevigata* | Rosaceae | No | No | – |
| *Rosa moschata* | Rosaceae | No | No | – |
| *Rosa multiflora* | Rosaceae | No | Yes | Yes |
| *Rosa nutkana* | Rosaceae | No | No | – |
| *Rosa rubiginosa* var*. rubiginosa* | Rosaceae | No | Yes | No |
| *Rosa setigera* | Rosaceae | No | No | – |
| *Rosa* ×*centifolia* | Rosaceae | No | No | – |
| *Rosa* ×*damascena* | Rosaceae | No | No | – |
| *Rosa* ×*noisettiana* | Rosaceae | No | No | – |
| *Rosa* ×*odorata* | Rosaceae | No | No | – |
| *Rubus plicatus* | Rosaceae | No | No | – |
| *Rudbeckia fulgida* | Asteraceae | Yes | Yes | No |
| *Rudbeckia hirta* | Asteraceae | Yes | Yes | Yes |
| *Rudbeckia triloba* | Asteraceae | Yes | Yes | No |
| *Ruta graveolens* | Rutaceae | Yes | Yes | No |
| *Sabatia campestris* | Gentianaceae | No | No | – |
| *Sagittaria sagittifolia* | Alismataceae | Yes | No | – |
| *Salix alba* | Salicaceae | Yes | Yes | Yes |
| *Salix babylonica* | Salicaceae | Yes | No | – |
| *Salix caprea* | Salicaceae | Yes | No | – |
| *Salix rosmarinifolia* | Salicaceae | No | No | – |
| *Salix* ×*forbyana* | Salicaceae | No | No | – |
| *Salix* ×*tetrapla* | Salicaceae | No | No | – |
| *Salpiglossis sinuata* | Solanaceae | Yes | No | – |
| *Salvia austriaca* | Lamiaceae | No | No | – |
| *Salvia azurea* | Lamiaceae | No | No | – |
| *Salvia coccinea* | Lamiaceae | Yes | No | – |
| *Salvia roemeriana* | Lamiaceae | No | No | – |
| *Salvia splendens* | Lamiaceae | Yes | No | – |
| *Salvia viridis* | Lamiaceae | No | No | – |
| *Sambucus nigra* | Caprifoliaceae | Yes | No | – |
| *Sanvitalia procumbens* | Asteraceae | Yes | No | – |
| *Saponaria calabrica* | Caryophyllaceae | No | No | – |
| *Saponaria ocymoides* | Caryophyllaceae | Yes | No | – |
| *Saponaria officinalis* | Caryophyllaceae | Yes | Yes | Yes |
| *Scabiosa atropurpurea* | Caprifoliaceae | No | Yes | No |
| *Schizanthus grahamii* | Solanaceae | No | No | – |
| *Scorpiurus vermiculatus* | Fabaceae | No | No | – |
| *Scyphanthus elegans* | Loasaceae | No | No | – |
| *Securigera varia* | Fabaceae | No | Yes | No |
| *Sedum acre* | Crassulaceae | Yes | Yes | Yes |
| *Sedum populifolium* | Crassulaceae | No | No | – |
| *Sedum pusillum* | Crassulaceae | No | No | – |
| *Sedum ternatum* | Crassulaceae | No | No | – |
| *Sempervivum tectorum* | Crassulaceae | Yes | Yes | No |
| *Senecio elegans* | Asteraceae | No | No | – |
| *Senna marilandica* | Fabaceae | Yes | No | – |
| *Sesbania punicea* | Fabaceae | No | No | – |
| *Sibiraea laevigata* | Rosaceae | No | No | – |
| *Sida hermaphrodita* | Malvaceae | No | No | – |
| *Silene armeria* | Caryophyllaceae | No | Yes | No |
| *Silene chalcedonica* | Caryophyllaceae | Yes | Yes | No |
| *Silene coeli-rosa* | Caryophyllaceae | No | No | – |
| *Silene coronaria* | Caryophyllaceae | Yes | Yes | No |
| *Silene dioica* | Caryophyllaceae | No | Yes | No |
| *Silene flos-cuculi* | Caryophyllaceae | Yes | Yes | Yes |
| *Silene flos-jovis* | Caryophyllaceae | No | No | – |
| *Silene fulgens* | Caryophyllaceae | No | No | – |
| *Silene noctiflora* | Caryophyllaceae | No | Yes | Yes |
| *Silene pendula* | Caryophyllaceae | No | No | – |
| *Silene schafta* | Caryophyllaceae | Yes | No | – |
| *Solanum chrysotrichum* | Solanaceae | No | No | – |
| *Solanum dulcamara* | Solanaceae | No | Yes | Yes |
| *Solanum macrocarpon* | Solanaceae | No | No | – |
| *Solanum marginatum* | Solanaceae | No | No | – |
| *Solanum robustum* | Solanaceae | No | No | – |
| *Sorbus aucuparia* | Rosaceae | Yes | Yes | Yes |
| *Sorbus domestica* | Rosaceae | No | No | – |
| *Sorbus* ×*thuringiaca* | Rosaceae | No | No | – |
| *Spiraea hypericifolia* | Rosaceae | No | No | – |
| *Spiraea japonica* | Rosaceae | Yes | Yes | No |
| *Spiraea salicifolia* | Rosaceae | Yes | No | – |
| *Spiraea thunbergii* | Rosaceae | Yes | No | – |
| *Spiraea vanhouttei* | Rosaceae | Yes | Yes | No |
| *Stachys byzantina* | Lamiaceae | Yes | Yes | No |
| *Stachys macrantha* | Lamiaceae | Yes | No | – |
| *Stachys pradica* | Lamiaceae | No | No | – |
| *Stictocardia tiliifolia* | Convolvulaceae | No | No | – |
| *Stipa pennata* | Poaceae | No | No | – |
| *Symphoricarpos orbiculatus* | Caprifoliaceae | Yes | No | – |
| *Symphytum asperum* | Boraginaceae | No | Yes | No |
| *Symphytum officinale* | Boraginaceae | Yes | Yes | Yes |
| *Syringa vulgaris* | Oleaceae | Yes | Yes | No |
| *Syringa* ×*persica* | Oleaceae | Yes | No | – |
| *Tagetes erecta* | Asteraceae | Yes | Yes | No |
| *Tanacetum coccineum* | Asteraceae | Yes | No | – |
| *Tanacetum parthenium* | Asteraceae | Yes | Yes | No |
| *Taxodium distichum* | Cupressaceae | No | No | – |
| *Taxus baccata* | Taxaceae | No | No | – |
| *Telekia speciosa* | Asteraceae | Yes | No | – |
| *Thalictrum thalictroides* | Ranunculaceae | No | No | – |
| *Thelesperma burridgeanum* | Asteraceae | No | No | – |
| *Thunbergia alata* | Acanthaceae | Yes | No | – |
| *Thymus vulgaris* | Lamiaceae | Yes | No | – |
| *Tilia platyphyllos* | Malvaceae | No | Yes | No |
| *Torenia fournieri* | Linderniaceae | Yes | No | – |
| *Tradescantia virginiana* | Commelinaceae | No | Yes | No |
| *Trichosanthes cucumerina* | Cucurbitaceae | No | No | – |
| *Trifolium rubens* | Fabaceae | Yes | No | – |
| *Trigonella caerulea* | Fabaceae | No | No | – |
| *Tripidium ravennae* subsp*. ravennae* | Poaceae | No | No | – |
| *Trollius europaeus* | Ranunculaceae | Yes | No | – |
| *Tropaeolum majus* | Tropaeolaceae | Yes | No | – |
| *Tropaeolum minus* | Tropaeolaceae | No | No | – |
| *Tropaeolum peltophorum* | Tropaeolaceae | No | No | – |
| *Tropaeolum peregrinum* | Tropaeolaceae | Yes | No | – |
| *Ulex europaeus* | Fabaceae | No | No | – |
| *Valeriana officinalis* | Caprifoliaceae | Yes | Yes | Yes |
| *Verbascum nigrum* | Scrophulariaceae | No | No | – |
| *Verbascum thapsus* | Scrophulariaceae | No | Yes | Yes |
| *Veronica spicata* | Plantaginaceae | Yes | Yes | No |
| *Veronica syriaca* | Plantaginaceae | No | No | – |
| *Veronicastrum virginicum* | Plantaginaceae | Yes | No | – |
| *Viburnum lantana* | Caprifoliaceae | Yes | Yes | No |
| *Viburnum opulus* var*. opulus* | Caprifoliaceae | Yes | Yes | No |
| *Viburnum plicatum* | Caprifoliaceae | Yes | No | – |
| *Vinca minor* | Apocynaceae | Yes | Yes | Yes |
| *Viola canina* | Violaceae | No | No | – |
| *Viola cornuta* | Violaceae | Yes | No | – |
| *Viola odorata* | Violaceae | Yes | Yes | No |
| *Viola tricolor* | Violaceae | Yes | Yes | Yes |
| *Wahlenbergia procumbens* | Campanulaceae | No | No | – |
| *Weigela hortensis* | Caprifoliaceae | No | No | – |
| *Wigandia urens* var*. caracasana* | Boraginaceae | No | No | – |
| *Wisteria frutescens* | Fabaceae | No | No | – |
| *Wisteria sinensis* | Fabaceae | Yes | No | – |
| *Xanthorhiza simplicissima* | Ranunculaceae | No | No | – |
| *Xeranthemum annuum* | Asteraceae | No | No | – |
| *Yucca filamentosa* | Asparagaceae | Yes | No | – |
| *Yucca flaccida* | Asparagaceae | Yes | No | – |
| *Zaluzianskya villosa* | Scrophulariaceae | No | No | – |
| *Zinnia violacea* | Asteraceae | No | No | – |
